# GPX1 expression promotes stemness and aggressiveness in myxoid liposarcomas

**DOI:** 10.7150/ijbs.105217

**Published:** 2025-08-30

**Authors:** Carmen Huergo, Juan Tornín, Oscar Estupiñán, Paula Díez, Borja Gallego, Jun Gao, Marybeth Creskey, Gauri Muradia, M Victoria González, Dzohara Murillo, Verónica Rey, Lucia Martínez-Cruzado, Sofía T. Menéndez, Mar Rodríguez‑Santamaría, Verónica Blanco, Isabel Quirós, Rosa M. Sáinz, Michael Rosu-Myles, Jessie R. Lavoie, René Rodríguez

**Affiliations:** 1 Instituto de Investigación Sanitaria del Principado de Asturias (ISPA), Hospital Universitario Central de Asturias, 33011 Oviedo, Spain.; 2 Instituto Universitario de Oncología del Principado de Asturias (IUOPA), 33011 Oviedo, Spain.; 3 Tumor Biology Research Group, Institute for Medical Biology, The Artic University of Norway, 9037 Tromsø, Norway.; 4 Centre for Oncology, Radiopharmaceuticals and Research, Biologic and Radiopharmaceutical Drugs Directorate, Health Products and Food Branch, Health Canada, Ottawa, ON, K1A 0K9, Canada.; 5 CIBER en oncología (CIBERONC), 28029 Madrid, Spain.; 6 Departamento de Cirugía, Universidad de Oviedo, 33006 Oviedo, Spain.; 7 Department of Pathology, University Hospital of Asturias (HUCA), Oviedo, Spain.; 8 Departamento de Morfología y Biología Celular, Universidad de Oviedo, 33006, Oviedo, Spain.; 9 Department of Biochemistry, Microbiology and Immunology, Faculty of Medicine, University of Ottawa, 451 Smyth Road, Ottawa, ON, K1H 8M5, Canada.

**Keywords:** GPX1, sarcoma, myxoid liposarcoma, cancer stem cells, oxidative stress, proteomics

## Abstract

The sarcomagenic process initiates when mesenchymal stromal/stem cells (MSCs) or MSC-derived cells undergo tumoral transformation. Besides, sarcoma evolution is partly driven by the emergence of subpopulations of cancer stem cells (CSCs), which are strongly associated with more aggressive behaviors. Therefore, the characterization of CSC will contribute to the development of more effective therapies against sarcomas. Here, we compared the proteomes of adherent and CSC-enriched tumorsphere cultures in a tumor progression model of myxoid liposarcoma composed of three cell lines showing increasing aggressiveness after being serially transplanted in mice. We found that the expression of the antioxidant enzyme GPX1 increased constantly during the CSC-enrichment process in this model and other sarcoma lines. Depletion of GPX1 resulted in decreased proliferation and tumorsphere-forming potential and dramatically reduced tumor-formation ability *in vivo*. Conversely, GPX1 overexpression resulted in increased proliferation and tumorsphere formation. According to these findings, GPX1 expression in sarcoma patients was associated with aggressive phenotypes and worse prognosis. A proteomic analysis revealed that these effects were associated with the downregulation of interferon-mediated response, the IL6/JAK/STAT3 axis and the NFκB-mediated signaling in GPX1-silenced cells. Overall, these results suggest that GPX1 expression may serve as a functional marker of aggressive CSC subpopulations in sarcomas.

## Introduction

Sarcomas are a group of rare malignancies that originate from mesenchymal tissues 1. Although they account for a relatively small proportion of all cancers, sarcomas exhibit significant inter- and intra-tumor heterogeneity and are often associated with poor prognosis 2, 3. Intratumor heterogeneity is in part due to a hierarchical organization of tumor cells sustained by subpopulations of tumor cells presenting stem cell properties 4. Similar to normal stem cells, these cancer stem cells (CSCs) display a drug-resistant phenotype and are responsible for relapses and tumor dissemination 5, 6. Therefore, a better understanding of specific pro-tumor signaling in CSCs is expected to lead to more efficient therapies for sarcomas.

A relevant protective functional feature of normal stem cells and CSCs is their tight control of reactive oxygen species (ROS) to prevent the harmful effects of oxidative stress 7. While ROS formation has a crucial role in normal cellular homeostasis, excess ROS and chronic oxidative stress can produce DNA damage leading to the emergence of deleterious mutations and the activation of oncogenes and pro-tumoral signaling pathways 8. Thus, multiple studies have confirmed a strong relationship between oxidative stress and the initiation and progression of several types of cancer 9, 10. However, ROS have a dual role in cancer and a dramatic increase in ROS levels induced by chemotherapy or radiation therapy can overwhelm the anti-oxidant defenses of cancer cells, thus triggering cell death 9, 11. In any case, these treatments are usually well suited for bulk tumor and highly proliferative cells, but CSCs often escape cell lethality induced by oxidative therapies in part by upregulating key antioxidant and detoxification pathways that neutralize ROS 7, 12.

Cell antioxidant defense systems rely on the activity of antioxidant enzymes such as superoxide dismutases, catalases, glutathione peroxidases (GPXs), and peroxiredoxins 9. GPXs are a family of eight antioxidant enzymes with peroxidase activity that catalyze the reduction of hydrogen peroxide and lipid hydroperoxides by converting glutathione to oxidized glutathione 13. Among them, the selenoprotein GPX1 is the most abundant isoform and is ubiquitously expressed in all cells, mainly located in the cytoplasm, mitochondria, nucleus, and peroxisomes 14. Aberrant expression of GPX1 in multiple cancers has been related to oncogenesis and cancer progression 13, 15. However, GPX1 has been reported to play both tumor suppressor and tumor promoter functions depending on the cancer type, the stage of the tumorigenic process and the influence of microenvironmental factors 9, 13. Thus, high expression of GPX1 was associated with adverse prognosis in acute myeloid leukemia, breast cancer or glioma 13, 16, 17; but more favorable prognosis in other types of cancer such as pancreatic cancer 18, 19. Among the pro-tumor features associated with GPX1, its expression was linked with increasing proliferation 13, 17, 20, 21, migration/invasion 13, 20, 22, 23, tumor growth 21, 24, and drug resistance 13, 20, 25, 26. Likewise, GPX1 activity was associated with the promotion of stemness properties. For instance, the regulation of the signaling axis microRNA-153/NRF2/GPX1 in glioma stem cells resulted in enhanced expression of GPX1 which was associated with neurosphere formation, stem cell marker expression and tumorigenic capacity 27. In another study, GPX1 was described as the most relevant antioxidant factor controlling the sensitivity of glioblastoma cells, including stem cell subpopulations, to oxidative stress 28. However, the specific role of GPX1 in sarcomas has not yet been addressed.

In this study, we performed a proteomic analysis of bulk monolayer cultures and sequential cultures of CSC-enriched tumorspheres of different cell lines capable of initiating myxoid liposarcomas (MLS) *in vivo* and which constitute a model of tumor evolution for this disease 29, 30. We identified GPX1 as one of the proteins that undergoes a more intense and gradual upregulation in CSC-enriched cultures. Gain and loss of function experiments and proteomic analyses confirmed the role of GPX1 in controlling the self-renewal, migration and tumor formation capacity of sarcoma cells. These results, coupled with the fact that GPX1 expression in sarcomas is correlated with poorer patient survival, suggest that this factor can serve as a functional marker of aggressive CSC subpopulations in sarcoma with clinically meaningful prognostic and therapeutic implications.

## Materials and Methods

### Cell culture and drugs

The MLS model (MSC-5H-FC, T-5H-FC#1 and T2-5H-FC#1 cell lines) was generated as previously described 30, 31. A brief description of the model is included in the results section. 1765-92 MLS (CVCL_S817) human MLS and 143-B (CRL-8303) human osteosarcoma cell lines were obtained from the ATCC repository (Manassas, USA). All cell lines were cultured as previously described 30, 32, and cultures were tested monthly to discard mycoplasma contamination using the Biotools Mycoplasma Gel Detection kit (B&M LABS, Madrid, Spain). Doxorubicin was purchased from Selleckchem (Cat# S1208 Houston, USA). Mercaptosuccinic acid (MSA) was purchased from Sigma (Cat#M6182; St Louis, USA). Stocks were prepared as 10 mM solutions in sterile DMSO, stored at -80 ºC and diluted in culture medium to the final concentration just before use.

### Cell viability assays

Cell viability of cell lines after the treatment with increasing concentrations of doxorubicin for 72 h was assayed using the cell proliferation reagent WST-1 (Roche, Mannheim, Germany) as previously reported 33.

### Real-time proliferation

Real-time cell proliferation was evaluated by loading 1 x 10^4^ cells in 8-well plates (E-plate L8) of the iCELLigence real-time cell analyzer (ACEA Biosciences, San Diego, USA) according to previously reported protocols 34. Cell impedance data was collected and analyzed using the RTCA Data Analysis Software 1.0 and cell proliferation was expressed as Cell Index (CI) normalized to the values measured 4 h after seeding.

### Colony Formation Unit (CFU) Assay

In CFU assays, 1 x 10^3^ T-5H-FC#1 or 1765-92 cells were plated in 100 mm culture dishes and allowed to grow for 10 days until isolated colonies were observed. The were cultures then fixed with cold methanol and stained with 0.5% crystal violet. Finally, colonies containing approximately more than 50 cells were scored using the ImageJ 2.1.0 software (NIH, Bethesda, USA).

### Migration assays

Transwell migration assay was performed and quantified as previously described 35. Briefly, 5 x 10^4^ 1765-92 cells suspended in serum-free culture media were plated in the upper chamber of transwell inserts (Costar, NY, USA) placed in 24-well plates containing 600 µL of medium (lower chamber). Following 24 h, cells were fixed in 70% ethanol for 10 min and stained with 0.5% crystal violet to assess their ability to migrate to the lower chamber. Before quantifying migration, those cells remaining on the upper side of the inserts were removed and the inserts were carefully washed with distilled water. Finally, the area of the membrane stained with crystal violet migrated cells was quantified using the ImageJ 2.1.0 software (National Institutes of Health, Bethesda, MD, USA).

### Tumorsphere culture

The tumorsphere culture protocol and quantification of tumorsphere formation were previously described 29, 33. Briefly, 5000 cells of any of the cell lines included in the study were seeded in 6-well low-attachment plates (Corning, NY, USA) and cultured in Ham's-F12 medium (Gibco, CA, USA) supplemented with B-27 Supplement (1:50; Gibco), 20 ng/ml EGF (PeproTech, London, UK), 10 ng/ml bFGF (PeproTech), Glutamax (Gibco) and Heparin (1:1000; Sigma-Aldrich, MO, USA). After 12 days of culture, tumorspheres were disaggregated with trypsin (0.25%)/EDTA (Gibco) and seeded for subsequent passages as described above. In some passages a subset of cultures was counted, and their cell viability was analyzed using the Cell Proliferation reagent WST-1 (Roche).

### Lentiviral constructions and cell transduction

Depletion of GPX1 was achieved using two lentiviral shRNA SMART vectors (Horizon Discovery Biosciences Limited, Cambridge, UK) targeted against two different sequences of GPX1, shGPX1 ID-05 (Clone ID: V3SVHS07_6205961), antisense: AACAGGACCAGCACCCATC, and shGPX1 ID-06 (Clone ID: V3SVHS07_5483591), antisense: TCCCGCAGGAAGGCGAAGA. A non-target shRNA was used as a negative control (pLemiR-NS, RRID: Addgene_32809, Addgene, Teddington, UK). In addition, we used a pLOC lentivirus vector (Precision LentiORF Collection; Horizon Discovery Biosciences Limited, Cambridge, UK) to overexpress GPX1 in 1765-92 MLS cells. The generation of lentiviral particles was performed as previously described 36. Transduced cells were positively selected through treatment with 20 µg/mL puromycin (Gibco, Carlsbad, USA) for 24 hours in the case of shRNA-carrying cell lines, and with 30 µg/mL blasticidin (Gibco) for 6 days in the case of cDNA cell lines.

### Proteomic analysis of CSC-associated factors

*Sample preparation.* Monolayer and first- and third-passage tumorsphere cultures of MSC-5H-FC, T-5H-FC#1 and T2-5H-FC#1 cells were gently harvested without trypsinization. Total protein extracts were prepared using RIPA buffer, and protein concentrations were determined by BCA assay (Thermo Fisher, Waltham, USA). A total of 100 ug/sample was reduced with 10 mM TCEP for 1 hr at 55 ºC and alkylated with 17 mM iodoacetamide for 30 min at RT in the dark. Then, proteins were digested with trypsin (1:25 enzyme:protein ratio) overnight at 37 ºC. Following these steps, peptides were labelled with TMT 10plex reagents (Thermo Fisher). Two experiments were combined. In each experiment, TMT-labelled peptides were quantified by colorimetric peptide assay (Thermo Fisher), combined at equal amounts an aliquot of each mixture was fractionated by either OFFgel IEF on the basis of isoelectric point or by high pH reversed phase. For experiment 1, the peptide mixture was subjected to high-resolution peptide OFFGel IEF fractionation (Agilent, Santa Clara, USA) pH 3-10 according to the manufacturer's instructions. Fractions were dried down and resuspended in 20 µL injection buffer containing 3% acetonitrile (MeCN), 0.2% formic acid (HCOOH), and 0.05% trifluoroacetic acid for analysis. For experiment 2, the peptide mixture was subjected to high pH reversed-phase peptide fractionation (Thermo Fisher) according to the manufacturer's instructions. Fractions were dried down and resuspended in 20 µl injection buffer containing 3% ACN, 0.2% FA, 0.05% TFA for analysis.

*Mass spectrometric analysis.* The Orbitrap Fusion Tribrid Mass Spectrometer coupled to an Easy-nLC 1000 (Thermo Fisher) was used to analyse fractionated and unfractionated the TMT labeled peptides mixture. The instrument was calibrated by infusion prior to analysis with a mixture of caffeine, MRFA, and Ultramark 1621. Two µL aliquots were analyzed by loading onto a NanoViper Acclaim pepmap 100 trap column (75 µm 20 mm with 3 µm beads) and desalting with 0.1% formic acid in water (solvent A) with 5 µL before separating on a NanoViper Acclaim pepmap RSLC C18 reverse-phase analytical column (50 µm 250 mm with 2 µm beads). Chromatographic separation was achieved at a flow rate of 0.300 µL/min over 240 min in seven linear steps as follows (solvent B was 0.1% formic acid in acetonitrile): initial, 5% B; 3 min, 5% B; 5 min, 10% B: 185 min, 25% B; 230 min, 60% B; 235 min, 90% B; 240 min, 90% B. The eluting peptides were analyzed in data-dependent mode for both MS2 and MS3 methods. A MS survey scan of 400-1500 m/z was performed in the Orbitrap at a resolution of 120,000 and an AGC target of 4 × 10^5^. The top speed mode was used to select ions for MS2 analysis, requiring charge state 2-7 and dynamic exclusion 40 s with a ± 10 ppm window, and monoisotopic precursor selection. During the MS2 analyses, precursors were fragmented by both collision-induced dissociation at 35% collision energy and by HCD at 30% stepped at 10%. Both fragmentation methods were followed by IonTrap analyses using automatic m/z normal scan range, precursors were isolated in the quadrupole using a width of 1.2, accumulated to an AGC target of 1 × 10^4^ or a maximum injection time of 50 ms. The 10 most intense fragments in MS2 spectra were selected for MS3 analysis with the filters mass range 400-1200, precursor exclusion +/- 5 m/z, and TMT isobaric tag loss exclusion. MS3 analysis was performed in the Orbitrap at resolution 60000 from 100-500 m/z, precursors isolated using a 2 m/z isolation window, accumulated to an AGC target of 5 × 10^4^ or a maximum injection time of 120 ms. The MS3 precursor population was isolated using the SPS waveform and then fragmented by HCD, with a normalized collision energy set to 65.

*Data processing*. The software package Proteome Discoverer 2.1 (Thermo Fisher) was used to process the data. The data from both experiments were combined. Two database search engines (SequestHT and Mascot) were used to search MS2 spectra against databases containing entries from human proteins (UniProt accessed on 20160401, 133803 entries) and common contaminants (cRAP). Fixed modifications were TMT tags on peptide N termini/lysine residues (+229.162932 Da) and carbamidomethylation of cysteine residues (+57.02146 Da) while variable modifications were N-terminal acetylation (+42.011 Da), methionine oxidation (+15.99492 Da) and serine, threonine, tyrosine phosphorylation (+79.966 Da). An MS2 spectra assignment false discovery rate (FDR) of less than 1% was achieved by applying the Percolator algorithm. For quantification using the MS3 spectra, a 20 ppm integration tolerance with the most confident centroid integration method was used. To account for differences in sample handling samples were normalized on the total peptide amount. Only MS3 spectra having a minimum average signal to noise (S/N) ratio of 20 were accepted for quantification.

### Proteomic analysis of GPX1-related signaling

*Sample preparation for LC-MS analysis.* Triplicated cultures of Sh-Control and ShGPX1-05 T-5H-FC#1 cells were harvested and processed for protein extraction. For each sample, 50 mg of protein was precipitated with 4 volumes of cold acetone and kept at -20 ºC for 2 h, followed by four pellet-washing steps with cold acetone. Samples were centrifuged at 20,000 g for 15 min, and the pellets were air-dried for 10 min and resuspended in RapiGest™ SF solution in 0.2% ammonium bicarbonate. Then, protein samples were incubated for 10 min at 40 ºC in agitation (400 rpm) followed by 5 cycles of sonication (90 sec ON/30 sec OFF) on ice. Once the sample solution became transparent, a total of 20 mg/sample was reduced with 5.5 mM DTT for 30 min at 60 ºC and alkylated with 12 mM iodoacetamide for 30 min at RT in the dark. Then, proteins were digested with trypsin/LysC (1:40 enzyme:protein ratio) overnight at 37 ºC. On the next day, digested samples were acidified with 0.5% TFA and incubated for 1 h at 37 ºC. Afterwards, peptide samples were centrifuged at 20,000 *g* for 10 min at 4 ºC and MeCN was added to the supernatant, followed by two extra centrifugation steps (20,000 *g* for 10 min) to remove any debris.

*LC-MS analysis and data processing.* For each sample, 400 ng of digested proteins were loaded on Evotips (Evosep) and were analyzed in a hybrid Q-TOF mass spectrometer (ZenoTOF 7600, Sciex, MA/USA) coupled to an Evosep One (Evosep, Odense, Denmark) liquid chromatography system. The peptide digests were separated using the 30 samples per day Evosep program (44 min total run time) with water and ACN, both with 0.1% HCOOH, as solvents A and B, respectively. Column temperature was set at 40 °C. An Optiflow electrospray ion source (Sciex) with a low-micro electrode was used for peptide ionization, applying a voltage of 4500 V and 100 °C. ZenoSWATH data-independent acquisition (DIA) was used as the MS method. This ZenoSWATH method consisted of cycles of one TOF MS scan (350 to 1250 m/z, 50 ms accumulation time) and 85 MS/MS of variable Q1 isolation windows (in the range 349.5 to 1247 m/z, acquired from 230 to 1400 m/z, 20 ms accumulation time, Zeno pulsing activated, dynamic collision energy). Data was acquired with the SciexOS software (Sciex). Automatic calibration at the TOF MS and MS/MS levels was performed after each sample with the X500 ESI Positive Calibration Solution (Sciex) using the calibrant delivery system of the mass spectrometer.

The ZenoSWATH runs were processed with DIA-NN v1.8.1 software using the library-free workflow according to the instructions from the authors. Thus, an *in silico*-predicted spectral library was built using the SwissProt database of human proteins (42,332 entries, including isoforms) and applied for the analysis of the ZenoSWATH runs. The main parameters used in DIA-NN were: 0 missed cleavages; N-terminal Met excision and Cys carbamidomethylation as fixed modifications; 2 to 5 precursor charge range; 350 to 1,500 precursor m/z range; 200 to 1,800 fragment ion m/z range; match-between-runs enabled; neural network classifier: double-pass mode; quantification strategy: robust LC (high precision); and RT-dependent cross-run normalisation. Protein groups were identified and quantified using only proteotypic peptides, and 1% FDR was used for both protein groups and precursors.

*Data analysis*. Count matrices from DIA-NN were analyzed using packages from R (4.3.2) and Bioconductor (3.18). Data were log-transformed and filtered out those proteins that were not identified at least in two of the three replicates for each condition. The remaining missing values were imputed by a maximum likelihood-based imputation method using the EM algorithm. Differential expression analysis was performed by applying empirical Bayes moderated t-statistics on protein-wise linear models using limma (3.58.1). Differentially expressed proteins (DEPs) were those with FDR ≤ 0.05 and |Log2FC| ≥ 0.5. Gene Set Enrichment Analysis (GSEA) was performed using clusterProfiler (4.10.0) and the MSigDB Hallmark collection (7.5.1). Transcription Factor activities were inferred using decoupleR (2.8.0) with the collecTRI collection and VIPER algorithm. Data visualization was performed using ggplot2 (3.5.0), ComplexHeatmap (4.3) and enrichplot (1.22.0).

### Availability of proteomic datasets

The mass spectrometry proteomics data have been deposited in the ProteomeXchange Consortium via the PRIDE partner repository with the dataset identifiers PXD053403 and PXD052595.

### Western Blotting

Whole-cell protein extraction and Western blot analysis were based on previously described protocols 35. Primary antibodies were as follows: anti-GPX1 (ab108427, 1:1000 dilution) from Abcam (Cambridge, UK); anti-IL6ST (67766-1-IG; 1:2000), anti-STAT1 (10144-2-AP; 1:1000), anti-SNAI1 (13099-1-AP; 1:1000) and anti-SNAI2/SLUG (12129-1-AP; 1:5000) from Proteintech (Manchester, UK); and anti-ß-actin (A5441, 1:5000) from Sigma. IRDye 800CW and IRDye-680RD from LI-COR Biosciences (1:10,000, Lincoln, USA) were used as secondary antibodies and fluorescent signals were detected using an Odyssey Fc imaging system and the Image Studio software (LI-COR Biosciences).

### Flow cytometry

*Cellular ROS detection (CellROX Assay)***.** The overall amount of reactive oxygen species (ROS) within cultures was measured with CellROX Deep Red (C10422; Thermo Fisher Scientific) fluorogenic probe and quantified by flow cytometry. Monolayer, CFU and tumorsphere cultures of control and GPX1 depleted 1765-92 cells were collected in polypropylene tubes and incubated with 5 µM of CellROX probe for 30 minutes. After this time, cells were pelleted and washed with PBS, and CellROX+ and CellROX- subpopulations were detected by flow cytometry using a Cytek Northern Lights (NL)-CLC spectral flow cytometer (Cytek Biosciences, Amsterdam, The Netherlands). Wild-type (non-transduced) 1765-92 cells were used as fluorescence negative control for gating purposes (Figure S1). Likewise, wild-type 1765-92 cells treated for 2 hours with culture medium containing 200 µM H2O2 were used as ROS positive control. Flow cytometry data were analyzed with the FlowJo v10.8 software (BD Biosciences, San Diego, California, USA).

*SOX2 levels.* SOX2 expression was detected by flow cytometry in cells fixed with paraformaldehyde (4%; 10 min at RT) and cold methanol (30 min at 4 ºC) using an anti-SOX2 primary antibody from Thermo Fisher (Waltham, MA) (PA1-094; 1: 1000 dilution) and an Alexa Fluor 647-conjugated secondary antibody from Thermo Fisher (A-21244; 2 µg/mL). A Normal Rabbit IgG (Thermo Fisher; 1:100) was used as control isotype.

### Xenograft experiments

Female 6 weeks old athymic nude mice (Envigo, Barcelona, Spain) were inoculated subcutaneously (s.c) with T-5H-FC or 1765-92 MLS transduced with either shCTRL, shGPX1-05 or shGPX1-06 lentiviral vectors. 5,000 and 50,000 cells suspended in medium and mixed 1:1 with BD Matrigel basement membrane matrix high concentration (Corning, NY, USA) previously diluted 1:1 in culture medium were inoculated in the right and left flanks respectively. Tumor size was measured with a caliper 2-3 times a week and tumor volume was determined using the equation (D × d^2^)/6 × 3.14, where D is the maximum diameter, and d is the minimum diameter, and values in each xenograft group were averaged. Animals were sacrificed by CO_2_ asphyxiation and tumors were extracted and weighed.

### Immunohistochemical Analysis

*Analysis of human sarcoma samples*. We used a tissue microarray containing 90 human sarcoma samples that were previously reported 32. Immunostaining of GPX1 was performed using an anti-GPX1 antibody (ab22604; dilution 1:200) from Abcam using the Dako EnVision Flex + Visualization System (Dako Autostainer, Denmark). Counterstaining with hematoxylin was the final step. A pathologist (VB) assessed the stained samples blindly, without access to clinical data, using a semiquantitative scoring system. The scoring was based on two factors: the percentage of stained cells (0: 0%; 1: <50%; 2: >50%) and the staining intensity (0: no expression; 1: low intensity; 2: high intensity). Each sample received a final score obtained by multiplying these two values. Based on this score, samples were classified as GPX1 negative (score = 0), GPX1 low (scores = 1-2), and GPX1 high (score = 4).

*Tumorspheres immunostaining*. Tumorsphere cultures of control and GPX-1 depleted 1765-92 were collected in 15 ml polypropylene tubes (Greiner Bio-One, Kremsmünster, Austria) and pelleted by gravity to avoid damage of spheres. Pellets were then washed twice in PBS, fixed with 4% formaldehyde and embedded in HistoGEL (HG-4000-012; Epredia, Kalamazoo, MI, USA) firstly and in paraffin afterwards for histological analyses. Immunohistochemistry detection of GPX1 was performed as described above for the analysis of tissue microarrays.

### Statistical analysis

Statistical analysis for both *in vitro* and *in vivo* experiments was conducted using GraphPad Prism software (GraphPad Software, Inc, La Jolla, CA, USA). Data are expressed as the mean (± standard deviation or SEM, as specified) from at least three independent experiments, unless stated otherwise. To assess statistical significance between groups, two-sided Student's t-tests or one-/two-way ANOVA tests were applied. For immunohistochemistry experiments, statistical analysis was performed using SPSS 24 software (SPSS, IBM Corp, Chicago, USA). The significance of differences among clinical groups was evaluated using the χ2 test, with Yates' correction when appropriate. Survival curves were generated using the Kaplan-Meier method, and differences in survival times were assessed with the log-rank test. A p-value of < 0.05 was considered statistically significant.

### Ethics approval

All experimental protocols involving human samples were conducted in accordance with institutional review board guidelines and the WMA Declaration of Helsinki. These protocols were approved by the Institutional Ethics Committee of the Principado de Asturias (ref. 255/19). Animal research protocols were pre-approved by the Animal Research Ethical Committee of the University of Oviedo (ref. PROAE 34-2019) before the study and were carried out in compliance with the institutional guidelines of the University of Oviedo.

## Results

### Proteomic analysis of a tumor evolution model identified CSC-associated markers in sarcomas

**Figure 1:**
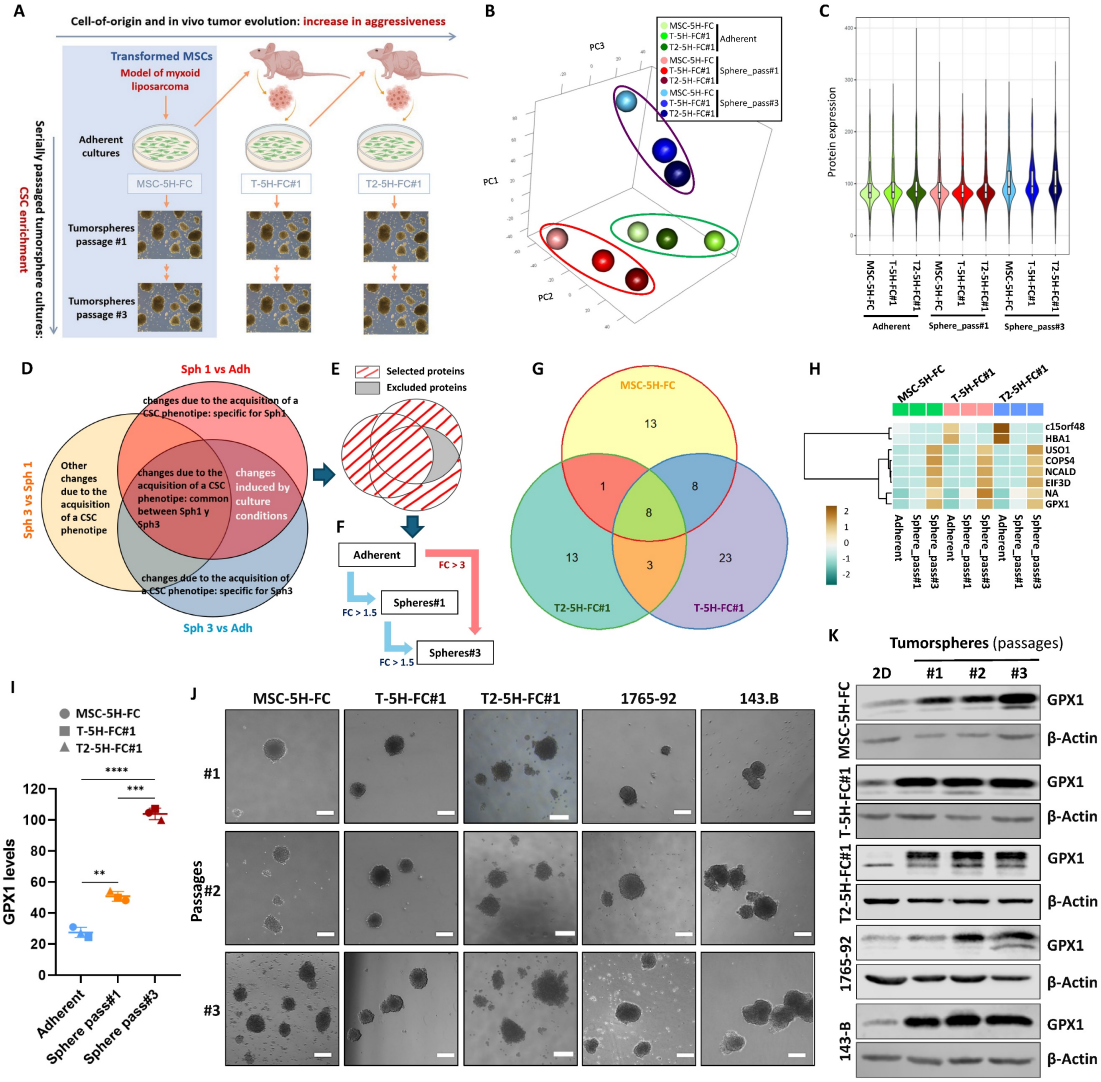
** Proteomic analysis of a sarcoma evolution model.** (A) Scheme of the generation of the MLS model. Mesenchymal stem cells transformed with six oncogenic hits including the FUS-CHOP (FC) fusion protein (MSC-5H-FC) were used as cells-of-origin of the sarcomagenic process. Cell lines derived after serial transplantation of MSC-5H-FC cells (T-5H-FC#1 and T2-5H-FC#1) displayed an increased aggressiveness phenotype (grey horizontal arrow). CSC-subpopulations in these models were enriched through sequential tumorsphere culture (grey vertical arrows). (B-I) proteomic analysis of bulk adherent cultures and cultures of tumorspheres at passages 1 and 3 in MSC-5H-FC, T-5H-FC#1 and T2-5H-FC#1 cells. (B) Principal component analysis of all samples. (C) Violin plot reflecting the global protein expression level of each variable. (D) Venn diagram showing the strategy followed to discard changes in protein expression due to the change in culture medium. (E) Diagram showing selected and excluded proteins. (F) Scheme with criteria for the selection of proteins of interest. (G) Venn diagram displaying the overlaps between proteins selected in MSC-5H-FC, T-5H-FC#1 and T2-5H-FC#1 cells. (H) Heat map showing commonly selected targets across all cell lines (NA: not identified peptide). (I) Levels of GPX1 detected in adherent cultures, passage 1 tumorspheres and passage 3 tumorspheres of all cell lines (**: p < 0.01;***: p < 0.001; ****:p < 0.0001; two-way ANOVA). (J) Representative images of tumorsphere cultures of the indicated cell lines in passages #1, #2 and #3 (scale bars = 200 µm). (K) Western blotting analysis of GPX1 in tumorsphere cultures of the indicated cell lines in passages #1, #2 and #3. β-actin was used as loading control.

We have formerly expressed the fusion oncogene FUS-CHOP, characteristic of MLS, on human MSCs previously transformed with five oncogenic hits (hTERT over-expression, p53 and Rb deficiency, c-myc stabilization and expression of H-RASv12; MSC-5H-FC cells) (Figure 1A) 30, 31. We have already shown that MSC-5H-FC cells were able to induce the formation of tumors resembling the main features of human MLS when inoculated into immunodeficient mice 30. The re-inoculation in subsequent recipients of serially established cell lines initially derived from an MSC-5H-FC-generated xenograft (T-5H-FC#1 and T2-5H-FC#1 cell lines) resulted in MLS formation after increasingly shorter latency periods 29, 30, therefore evidencing the existence of an MLS-CSC subpopulation. Moreover, MSC-5H-FC, T-5H-FC#1 and T2-5H-FC#1 cells could be serially expanded as clonal spheres floating cultures (tumorspheres) 29, a property associated with self-renewal and the anoikis resistance characteristic of CSCs 37, 38. Importantly, we previously demonstrated that the sphere-forming subpopulation of T-5H-FC#1 cells induces MLS formation in immunodeficient mice much more efficiently than the bulk adherent cultures, confirming that this subpopulation is enriched in CSCs 29. Also in this line, we previously shown that tumorsphere cultures of MSC-5H-FC, T-5H-FC#1 expressed enhanced levels of pluripotency factors like SOX2, and ALDH1A1 29, 36. Altogether, these results confirm the suitability of this collection of serially established cell lines and 3D cultures as a bidimensional model to study CSC-associated features related to both increased tumor aggressiveness (MSC-5H-FC < T-5H-FC#1 < T2-5H-FC#1) and stemness properties (adherent cultures < tumorsphere cultures) (Figure 1A).

To unravel new molecular mechanisms associated with the development of aggressiveness and stemness during the sarcomagenic process, we performed a proteomic analysis of bulk adherent cultures (Adh) and cultures of tumorspheres at passage 1 (Sph1) and 3 (Sph3) in MSC-5H-FC, T-5H-FC#1 and T2-5H-FC#1 cells (Figure 1B-H). First, a principal component analysis (PCA) showed that the samples cluster primarily based on cell culture type, with adherent cultures and first-passage tumorspheres displaying differences along PC3 and third-passage tumorspheres exhibiting greater differences in gene expression along PC1 (Figure 1B). In relation to these differences, we observed that the third-passage sphere cultures show higher levels of protein expression than the other cultures (Figure 1C). Within each culture type, the proteome profile of MSC-5H-FC, T-5H-FC#1 and T2-5H-FC#1 cells differed along PC2 (Figure 1B).

Next, we compared the protein expression levels among the different culture types (Sph1 vs Adh; Sph3 vs Adh; and Sph3 vs Sph1) for each cell line and selected those proteins with log2 (Fold Change) above 0.5 or below -0.5 (|LogFC| ≥ 0.5) for downstream analyses. Before further processing selected targets, it is necessary to take into account the influence that the differences in the culture conditions (monolayer vs tumorsphere cultures) may have on protein expression. To this end, we assumed that those changes due to variations in culture conditions should be shared by Sph1 vs Adh and Sph3 vs Adh comparisons. Besides, considering that stemness-related traits can be selected through sequential tumorsphere culture, we hypothesized that changes observed in Sph3 vs Sph1 were genuine stemness-related events. Therefore, to minimize the influence of changes in protein expression which may be derived from the different culture conditions, we discarded those targets that were altered in both the Sph1 and Sph3 cultures compared to the adherent cultures but did not change in the Sph3 vs Sph1 cultures (Figure 1D-E, Figure S2A-C). Of the remaining targets, and also assuming a sequential enrichment of CSC-related targets during tumorsphere culture, we selected those proteins that met one of the following criteria: 1) their expression increased or decreased constantly (Log2FC ≤-0.5 or ≥ 0.5) throughout all passages (Sph3 ≥ Sph1 ≥ Adh or Sph3 ≤ Sph1 ≤ Adh); or 2) showed at least a three-fold change (log2FC ≤-1.5 or ≥1.5) in expression between third-generation spheres and adherent cultures (Figure 1F, Figure S2A-C). Following these criteria, we selected 30 proteins in MSC-5H-FC (25 with an upregulatory trend and 5 downregulated), 42 in T-5H-FC#1 (29 upregulated and 13 downregulated) and 25 in T2-5H-FC#1 cells (19 upregulated and 6 downregulated) (Figure S2D-F, Tables S1-S3). To further define CSC-associated markers, we compared selected targets for each cell line. We found that MSC-5H-FC, T-5H-FC#1 and T2-5H-FC#1 cells share 8 proteins that were upregulated (6) or downregulated (2) during the selection of CSC-like cells (Figure 1G-H). Given that alterations in redox signaling may play pivotal roles both in tumorigenesis 8 and the maintenance of the stem phenotype 7, we selected the antioxidant enzyme GPX1 for further characterization. Besides an unidentified peptide (NA), GPX1 was the only one of the selected targets that meets the criterion '1'), displaying a continuous and significant upregulation from adherent cultures to sequentially passaged tumorspheres (Adh < Sph1 < Sph3) (Figure 1I, table S4). In any case, the levels of this factor remained stable across all cell types in the model at each passage level (Figure 1I). This suggests that while GPX1 may contribute to stemness and aggressiveness at each stage, it may not play a significant role in the progressive increase in aggressiveness observed throughout the evolution of the model (MSC-5H-FC < T-5H-FC#1 < T2-5H-FC#3).

To validate the overexpression of GPX1 in CSC subpopulations, we analyzed its expression by Western blotting in the three cell lines analyzed in the proteomic study, as well as in other MLS line (1765-92 MLS) and an osteosarcoma line (143B). In these experiments, we used extracts from adherent cultures and tumorsphere cultures from three successive generations (Figure 1J). In line with the results of the proteomic analysis, we observed that, compared to adherent cultures, GPX1 levels dramatically increase in the first-generation tumorsphere cultures in all models. Furthermore, a slight gradual increase in GPX1 expression is also observed during serial passages of tumorspheres in all assayed cell lines, except in 143-B cells, where a very high level of expression is already observed in 1st-passage turmorspheres (Figure 1K and Figure S3). This confirms that this target is overexpressed in CSC-enriched subpopulations in different sarcoma lines.

### GPX1 Expression in Sarcoma Tissue Specimens is Associated with Poor Prognosis and Survival

**Figure 2:**
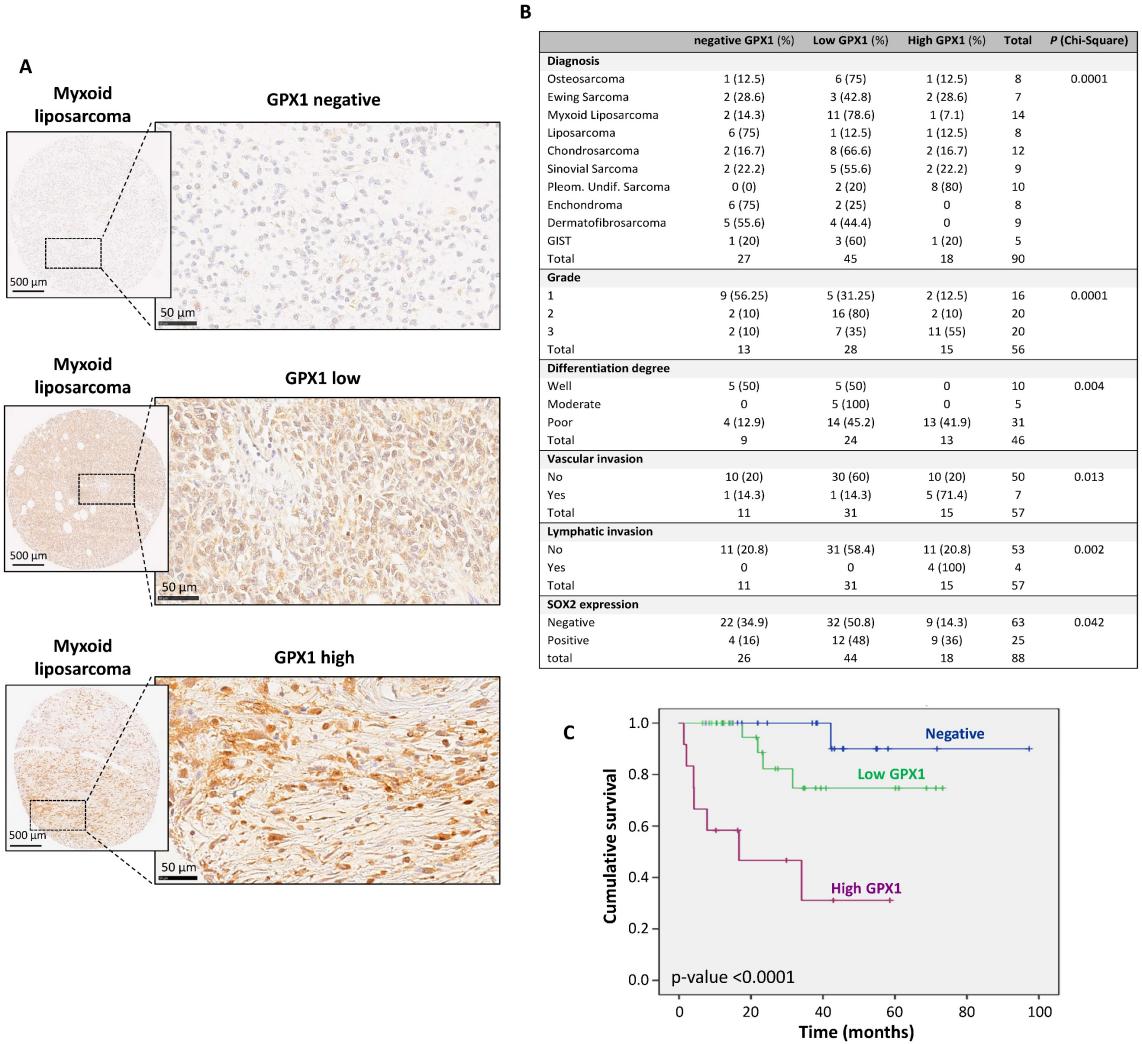
** Immunohistochemical analysis of GPX1 expression in a tissue microarray of human sarcoma samples and associations with clinical data.** (A) Representative examples of myxoid liposarcomas showing negative, low and high levels of GPX1 staining. Scale bars: 500 µM (left panels) and 50 µM (right panels). (B) Distribution of sarcoma cases (N = 90) according to their GPX1 expression level across categories of the indicated patient characteristics and tumor clinicopathologic parameters. Note that grade, differentiation degree, vascular and lymphatic invasion refer exclusively to malignant tumor cases and that all benign tumors exhibited negative/low GPX1 expression. p values of chi-square test are provided. (C) Kaplan-Meier cumulative survival curves categorized by GPX1 protein expression (negative, n=22; low, n=29; and high levels, n=12) in the cohort of sarcoma patients. p-values were estimated using the log-rank test. GIST: gastrointestinal stromal tumor.

To investigate whether GPX1 expression in sarcomas is clinically relevant, its expression was analyzed in a tissue microarray collection with 90 samples representing 10 types of sarcomas. In this immunohistochemical analysis, cytoplasmic expression of GPX1 was detected in 63 (70%) of them, with 45 showing low levels of expression and 18 showing elevated levels (Figure 2A-B). GPX1 expression significantly correlated with higher tumor grade (p = 0.0001), lower differentiation (p = 0.004), increased vascular invasiveness (p = 0.013), lymphatic invasiveness (p = 0.002) and higher levels of SOX2 (p = 0.042) (Figure 2B). Importantly, the level of GPX1 expression is inversely associated with the survival of sarcoma patients in a statistically significant way. The 5-year survival rate (60 months) is approximately 90% for negative cases and between 30% and 70% for positive cases (Figure 2C). These data suggest that GPX1 correlates with advanced tumor stages, more aggressive phenotypes, and a worse prognosis.

### Modulation of the expression of GPX1 influences the growth properties and migration capability of sarcoma cells

Given the relevant role that GPX1 might play in the sarcomagenic process, we generated loss- and gain-of-function models to investigate its potential role in pro-tumorigenic traits associated with increased aggressiveness.

**Figure 3:**
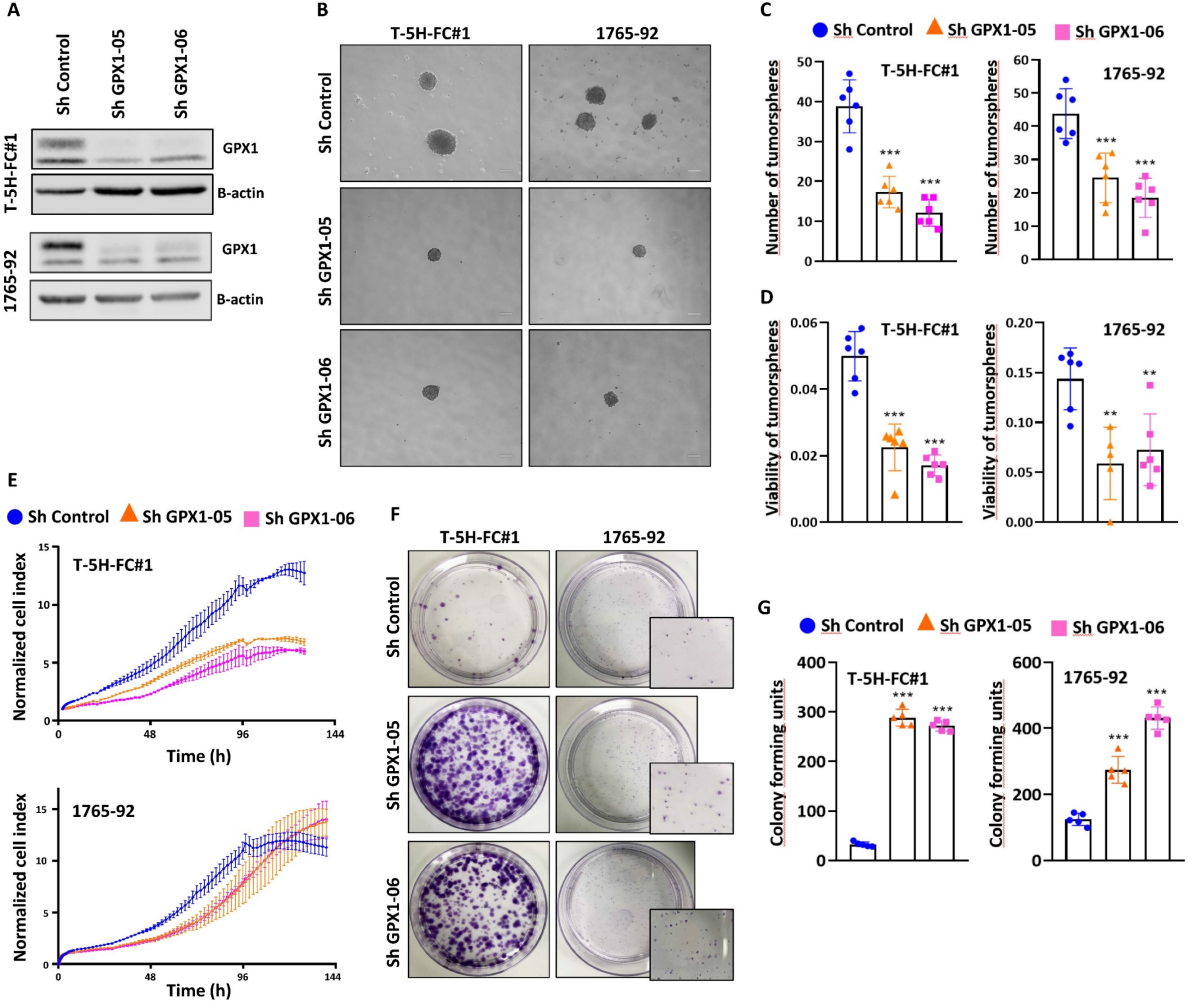
** The depletion of GPX1 reduced the proliferation and tumorsphere-forming potential of sarcoma cells**. (A) Protein expression levels of GPX1 in control (sh Control) and GPX1-silenced (sh GPX1-05 and shGPX1-06) T-5H-FC#1 and 1765-92 cells. β-actin was used as a loading control. (B-D) Tumorsphere-forming ability of control and GPX1-depleted cells. Representative images (scale bars = 200 µm) (B), and quantification of the number of tumorspheres (C) and cell viability (WST-1 assays) (D) are presented. (E) Real-time proliferation (cell index) of control and GPX1-depleted cells measured using an iCelligence system. (F-G) CFU assay of control and GPX1-silenced cells. Representative pictures (F) and quantification (G) of CFU assays for each cell line are shown. Data are presented as the mean and the standard deviation of at least three biological replicates. Asterisks indicate statistically significant differences (**: p < 0.01; ***: p < 0.001; one-way ANOVA).

**Figure 4:**
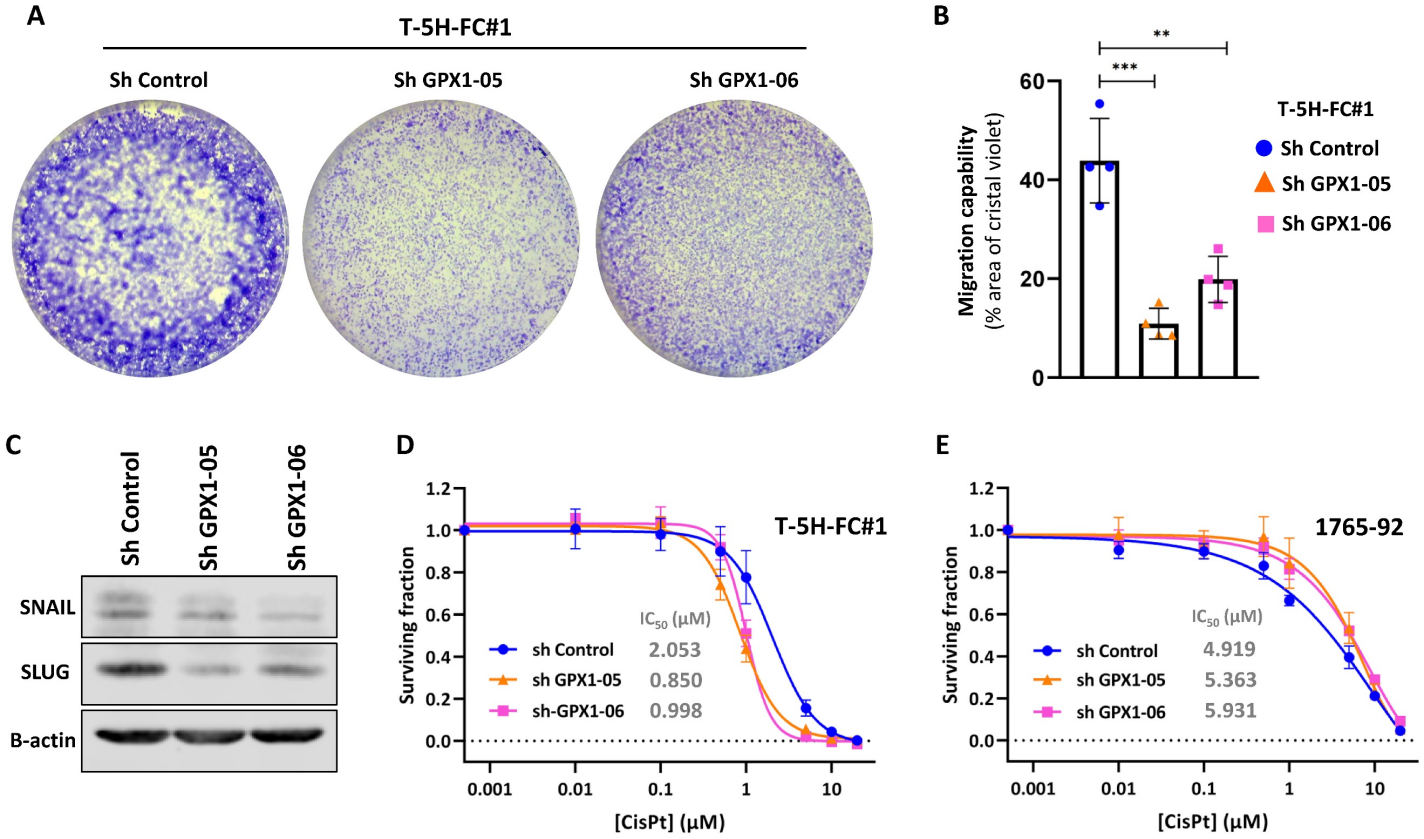
** Migration capability and drug response of GPX1-silenced sarcoma cells**. (A-B) Transwell migration assay of control (sh Control) and GPX-1 silenced (sh GPX1-05 and shGPX1-06) T-5H-FC#1 cells. Representative images (A) and quantification of the surface occupied by migrated cells (B) are shown. (C-D) Cell viability (WST-1 assays) was measured after the treatment of control and GPX1 depleted T-5H-FC#1 (C) and 1765-92 (D) cells with increasing concentrations of cisplatin for 72 h. IC_50_ values for cisplatin treatments are shown. Data represents the mean and SD of at least three independent experiments. Asterisks indicate statistically significant differences (**: p < 0.01; ***: p < 0.001; one-way ANOVA).

For these experiments, we focused on MLS models. First, we used two specific shRNA sequences (sh GPX1-05 and sh GPX1-06) to produce GPX1-depleted variants of T-5H-FC#1 and 1765-92 cell lines (Figure 3A). We found that all GPX1-depleted lines showed a significantly decreased ability to grow as tumorspheres compared to control cells, as observed by scoring the number of spheres formed or by measuring its viability (Figure 3B-D). This impaired tumorsphere-forming ability to form is associated to a reduction in SOX2 levels in GPX1-depleted cells (Figure S4) and is also observed during serial passaging of GPX1-silenced T-5H-FC#1 tumorspheres (Figure S5). Next, we used the iCelligence™ system to follow the real time proliferation of the different cultures. We found that the inhibition of GPX1 with both shRNAs in the two cell lines resulted in slower proliferation capacity (Figure 3E). However, despite this lower proliferative phenotype, GPX1-depleted cells showed a significantly enhanced colony formation capacity (Figure 3F-G). Furthermore, transwell migration assays showed that GPX1 depletion significantly reduced the migration capability of T-5H-FC#1 cells (Figure 4A-B), and this effect was associated with the downregulation of migration-promoting factors such as SNAIL and SLUG (Figure 4C and Figure S6). Finally, we found that GPX1 depletion in T-5H-FC#1 cells increased sensitivity to cisplatin by approximately two-fold (IC50: shControl = 2.053 µM, shGPX1-05 = 0.850 µM, shGPX1-06 = 0.998 µM) (Figure 4D), consistent with previous studies reporting that GPX1 contributes to resistance to this compound in other tumor types 20, 25. However, this moderate chemosensitizing effect appears to be cell type-dependent, as no enhanced response to cisplatin was observed in GPX1-silenced 1765-92 cells (Figure 4E). Furthermore, treatment with a different chemotherapeutic agent, doxorubicin, did not improve the response in either GPX1-depleted cell line (Figure S7).

**Figure 5:**
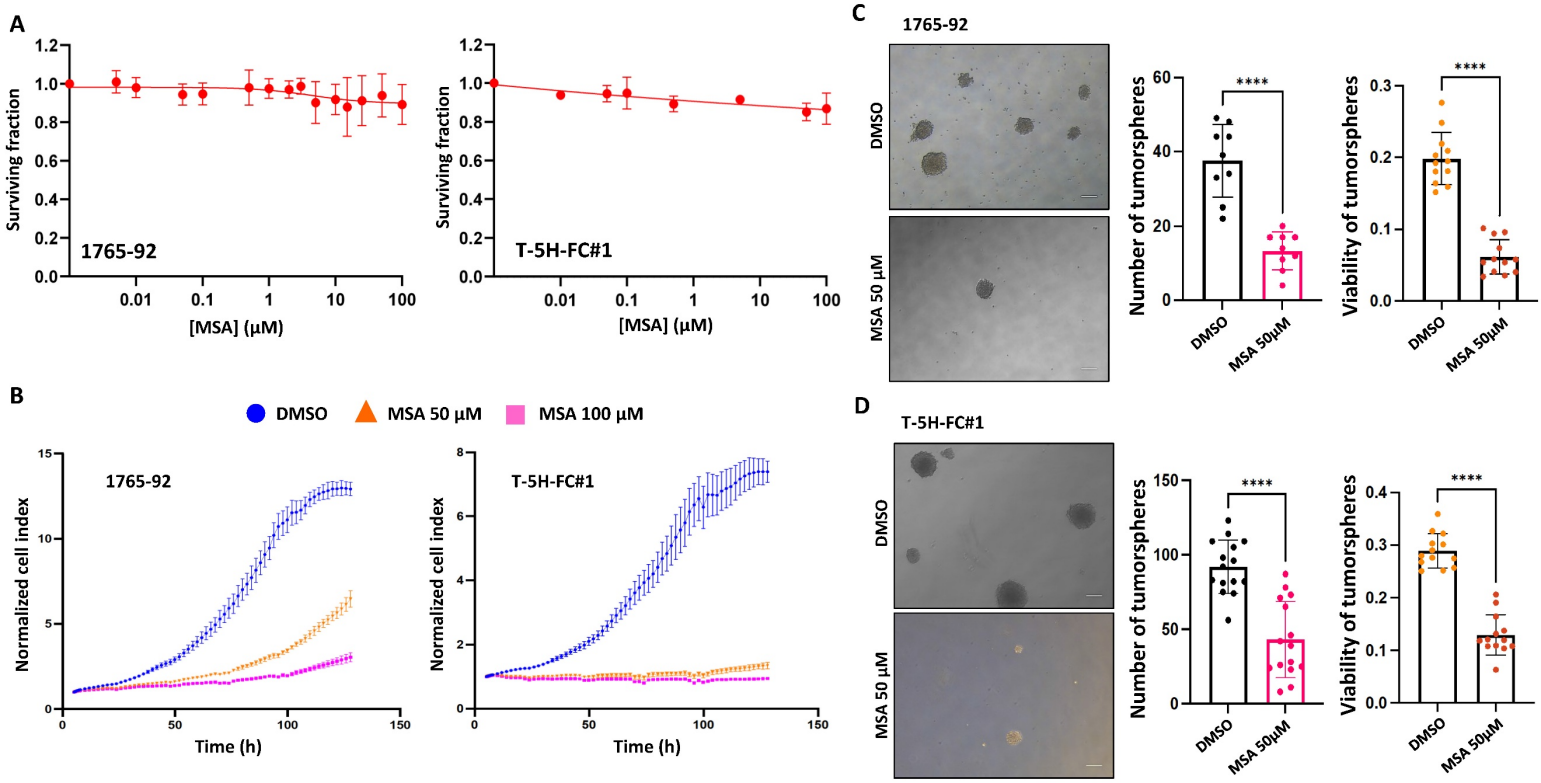
** Effect of MSA in the proliferation and tumosphere-forming potential of sarcoma cells.** (A) Cell viability (WST-1 assays) measured after the treatment of 1765-92 (left panel) and T-5H-FC#1 (right panel) cells with increasing concentrations of MSA for 72 h. (B) Real-time proliferation (cell index) of 1765-92 (left panel) and T-5H-FC#1 (right panel) cells treated with DMSO (vehicle), 50 µM or 100 µM MSA. (C-D) Tumorsphere-forming ability of control and GPX1-depleted 1765-92 (C) and T-5H-FC#1 (D) cells. Representative images (scale bars = 200 µm) (left panels), quantification of the number of tumorspheres (middle panels) and cell viability (right panels) are presented. Data are presented as the mean and the standard deviation of at least three biological replicates. Asterisks indicate statistically significant differences (****:p < 0.0001; two-sided Student *t* test).

Seeking further support for these findings, we studied the effect of the pharmacological inhibition of GPX1 using mercaptosuccinic acid (MSA), a well-known inhibitor of its enzymatic activity 28, 39. Even at high concentrations, MSA treatment was not toxic to 1765-92 and T-5H-FC#1 cells (Figure 5A). However, as observed in GPX1 depletion experiments, this compound greatly inhibited the proliferation (Figure 5B) and the tumorsphere-forming potential of these cell lines (Figure 5C-D). On the other hand, we did not observe any effect of MSA on the migratory ability of T-5H-FC#1 cells (Figure S8). In this regard, it may be of interest to investigate the effects of novel and more specific GPX1 inhibitors, such as members of the pentathiepin family, which have been reported to be up to 15 times more potent GPX1 inhibitors than MSA 40.

**Figure 6:**
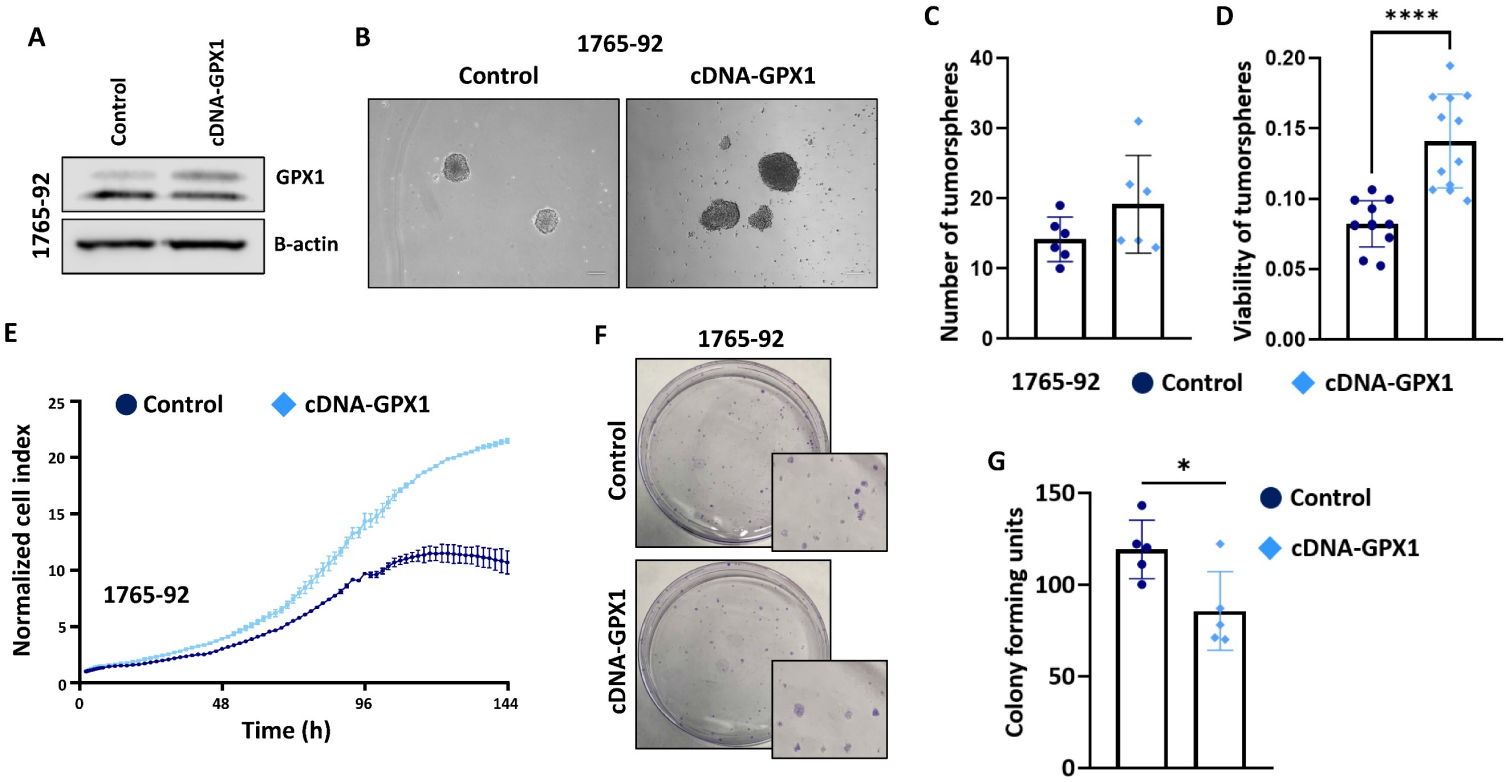
** The overexpression of GPX1 increased the proliferation and tumorsphere-forming potential of sarcoma cells.** (A) Protein expression levels of GPX1 in control (sh Control) and GPX1-overexpressing (cDNA) 1765-92 cells. β-actin was used as a loading control. (B-D) Tumorsphere-forming ability of control and GPX1-overexpressing cells. Representative images (scale bars = 200 µm) (B), and quantification of the number of tumorspheres (C) and cell viability (WST-1assays) (D) are presented. (E) Real-time proliferation (cell index) of control and GPX1-overexpressing cells measured using an iCelligence system. (F-G) CFU assay of control and GPX1 overexpressing cells. Representative pictures (F) and quantification (G) of CFU assays are shown. Data are presented as the mean and the standard deviation of at least three biological replicates. Asterisks indicate statistically significant differences (*: p < 0.05; ****: p < 0.0001; two-sided Student *t* test).

In full agreement with the results obtained with the genomic inhibition of GPX1, the overexpression of this antioxidant factor in 1765-92 cells (Figure 6A and Figure S9) resulted in increased tumorsphere formation (Figure 6B-D), increased proliferation potential (Figure 6E) and decreased clonogenic capacity (Figure 6F-G).

Collectively, these results suggest that GPX1 levels affect features related to the stemness potential and the proliferative and migrative capacity of sarcomas cells.

### Regulation of ROS in GPX1-Depleted Cells

**Figure 7:**
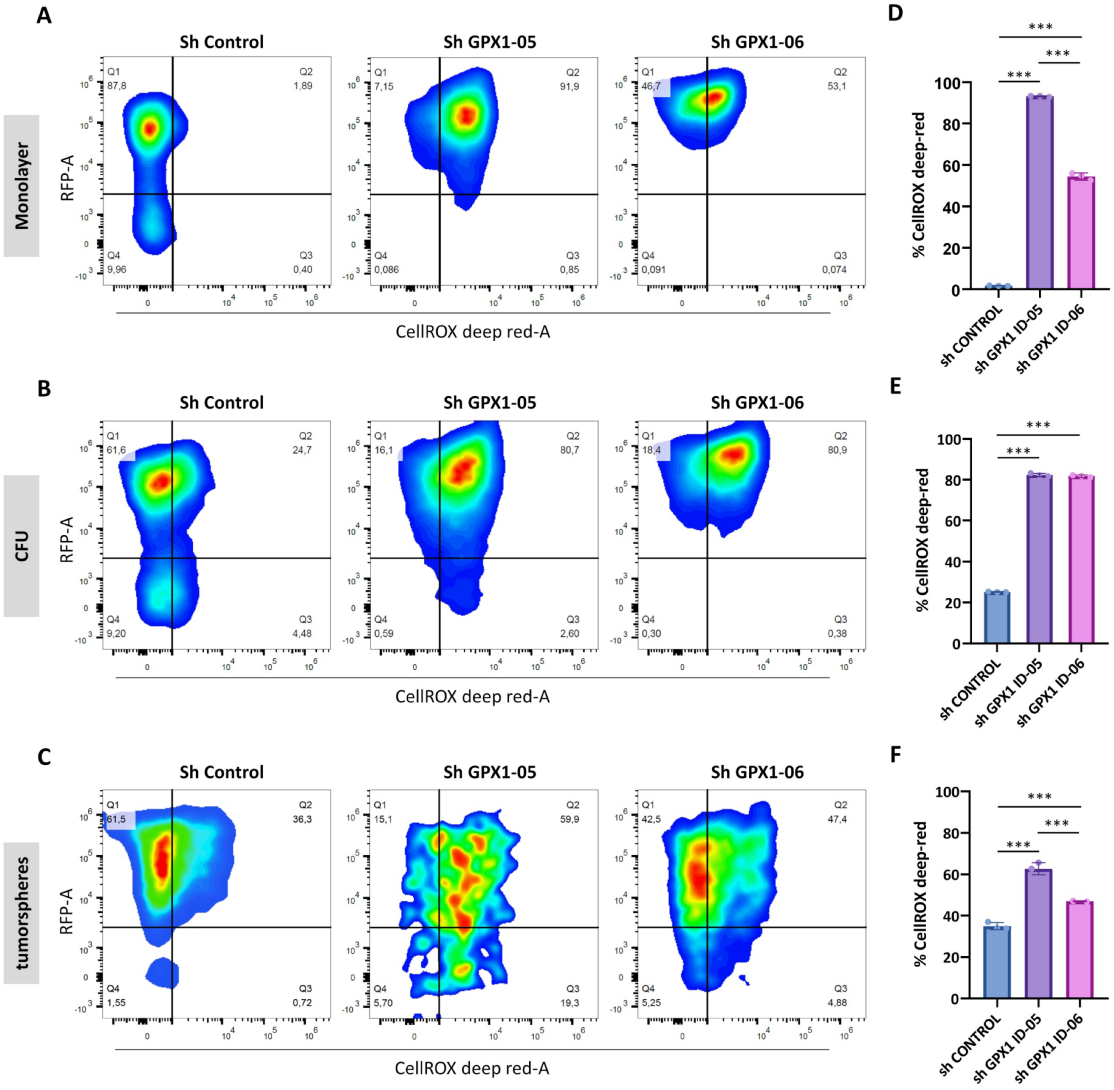
** Modulation of ROS levels in GPX1-depleted cells.** Quantification of ROS levels in Monolayer (A and D), CFUs (B and E) and tumorsphere cultures (C and F) of 1765-92 cells using the CellROX assay. Representative dot plots (RFP vs CellROS fluorescence) of flow cytometry analyses (A-C) and quantification of the percentage of CellROX in RFP positive cells (D-F) of three biological replicates for each type of culture are shown. The gating strategy, including negative and positive controls, is presented in Figure S1. Asterisks indicate statistically significant differences (***: p < 0.001; one-way ANOVA).

To assess the extent to which ROS regulation contributes to the distinct phenotypes observed in GPX1-depleted cells, we analyzed intracellular ROS levels in monolayer, CFU, and tumorsphere cultures of control and GPX1-silenced 1765-92 cells (Figure 7A-C). Interestingly, ROS levels varied depending on the culture type. Establishing the monolayer sh Control cells as a gating control, we found that the relative levels of ROS in control conditions followed the order: monolayer < CFUs < tumorspheres (Figure 7D-F). These findings suggest that different culture conditions select for distinct subpopulations. Specifically, CFU and tumorsphere cultures appear to enrich cell subsets that are adapted to grow under higher basal ROS levels.

Under GPX1-silenced conditions, all culture types displayed significantly elevated ROS levels compared to their respective controls (Figure 7D-F). This substantial increase in intracellular ROS may contribute to the inhibition of tumorigenic and stem-like properties in GPX1-depleted sarcoma cells.

### Proteomic analysis of GPX1-depleted cells

**Figure 8:**
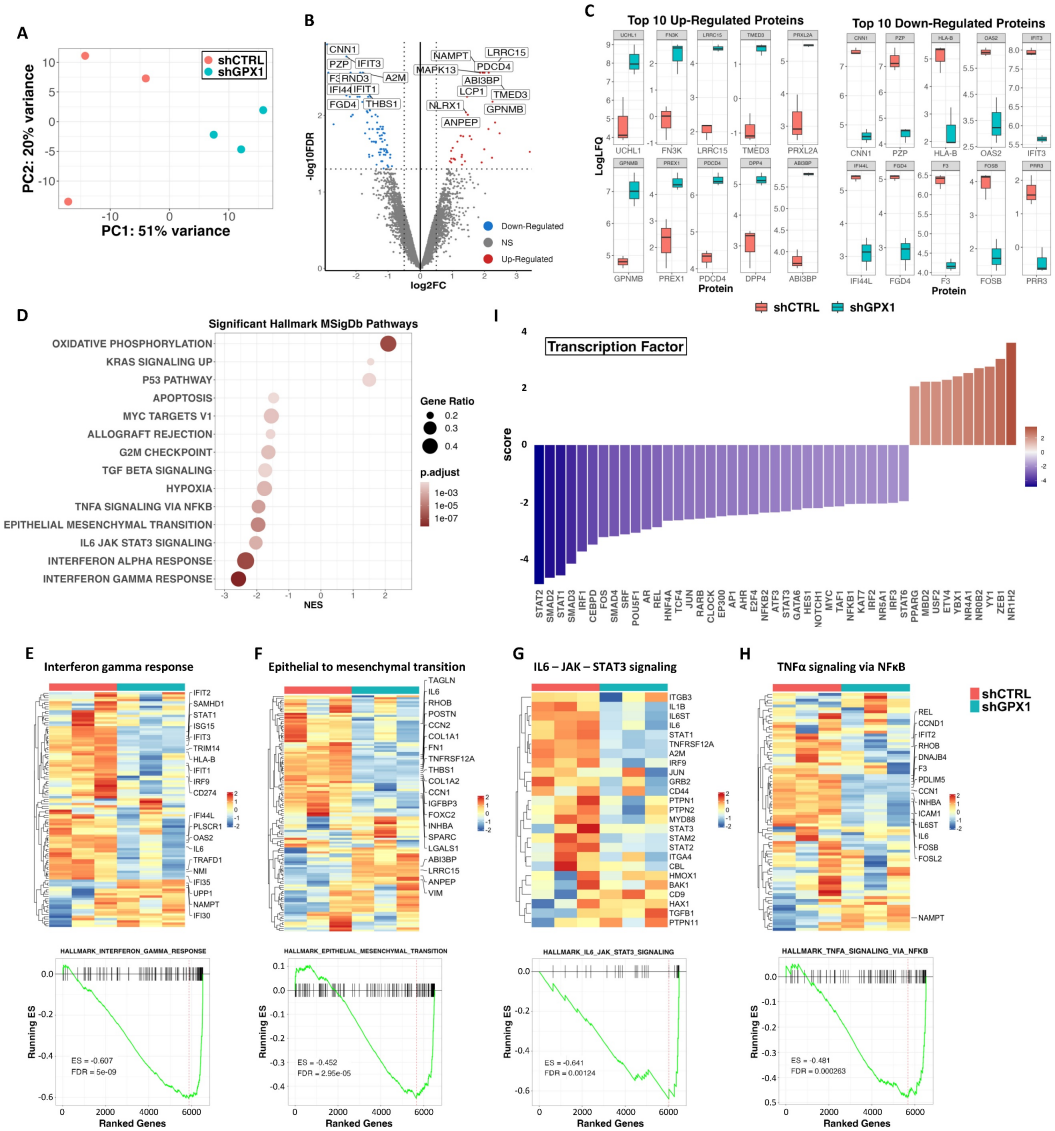
** Proteomic analysis of GPX1-depleted cells.** Triplicated monolayer cultures of control (shCTRL) and GPX1-depleted (shGPX1-05) T-5H-FC#1 cells were processed for proteomic analysis. (A) Principal component analysis of all samples. (B) Volcano plots showing those proteins significantly (FDR ≤ 0.05 and |LogFC| ≥ 0.5) up-regulated (red dots) and downregulated (blue dots) in shGPX1 vs shCTRL conditions. NS indicates not significant changes (grey dots). Selected proteins displaying highly significant p values and/or high fold change modulation are indicated. (C) Levels of the 10 top upregulated (left panels) and downregulated (right panels) proteins in shGPX1 vs shCTRL conditions. (D) Bubble plots showing significantly enriched pathways (GSEA, FDR <0.05) from the MSigDB Hallmark collection in shGPX1 vs shCTRL conditions. (E-H) Top panels: heatmap plots showing the expression values of those DEPs of the Hallmarks IFNγ response (E), epithelial to mesenchymal transition (F), IL6-JAK-STAT3 signaling (G) and TNFα signaling via NFκB (H). Bottom panels: GSEA analysis of these signaling pathways. Enrichment score (ES) and FDR values are indicated. (I) Inference of altered transcription factor-mediated signaling from differentially expressed proteins.

To gain insight into the molecular basis behind the anti-tumor effects observed after the inhibition of GPX1 in sarcoma cells, we performed a proteomic analysis in triplicate samples of control and GPX1-depleted (Sh GPX1-05) T-5H-FC#1 cells (Figure 8A). Comparing sh GPX1-05 vs sh Control conditions, we detected 116 DEPs (log2 (FC) ≤ -0.5 or ≥ 0.5 and padj < 0.05), with a higher proportion of targets downregulated (81) than upregulated (35) (Figure 8B and Table S5). Among the 10 most upregulated proteins in GPX1-depleted cells, UCHL1 has been linked to a stemness phenotype in different types of cancer 41, 42; FN3K and PRXL2 are involved in the regulation of redox signaling 43, 44; and other targets such as LRRC15 and TMED3 have been found overexpressed and/or related to tumorigenesis in sarcomas 45, 46 (Figure 8C). On the other hand, among the 10 most downregulated proteins, we found factors related to IFN signaling, such as IFIT3 or IFI44L, and transcription factors of the AP-1 complex, which has been previously related to the development of bone sarcomas from MSCs 47 (Figure 8C and Table S6). GSEA of DEPs also revealed a higher proportion of significantly downregulated vs upregulated pathways in GPX-1 depleted cells (Figure 8D and Table S7). Thus, interferon-mediated signaling (Figure 8E), the IL6/JAK/STAT3 axis (Figure 8F), the epithelial to mesenchymal transition (Figure 8G) and the NFκB-mediated signaling (Figure 8H) were significantly repressed in T-5H-FC#1 - sh GPX1-05 cells. The inference of altered transcription factor-mediated signaling from DEPs also indicated downregulation of NFκB signaling (negative scores for REL, NFκB2 and NFκB1), Interferon signaling (negative scores for STAT1, STAT2, IRF1 and IRF2), IL6 signaling (negative scores for STAT3 and MYC), AP1-mediated signaling (negative scores for FOS, JUN and AP1) and NOTCH signaling (negative scores for NOTCH1, and HES1) (Figure 8I).

### GPX1 is required to maintain the tumorigenic potential of sarcoma cells

**Figure 9:**
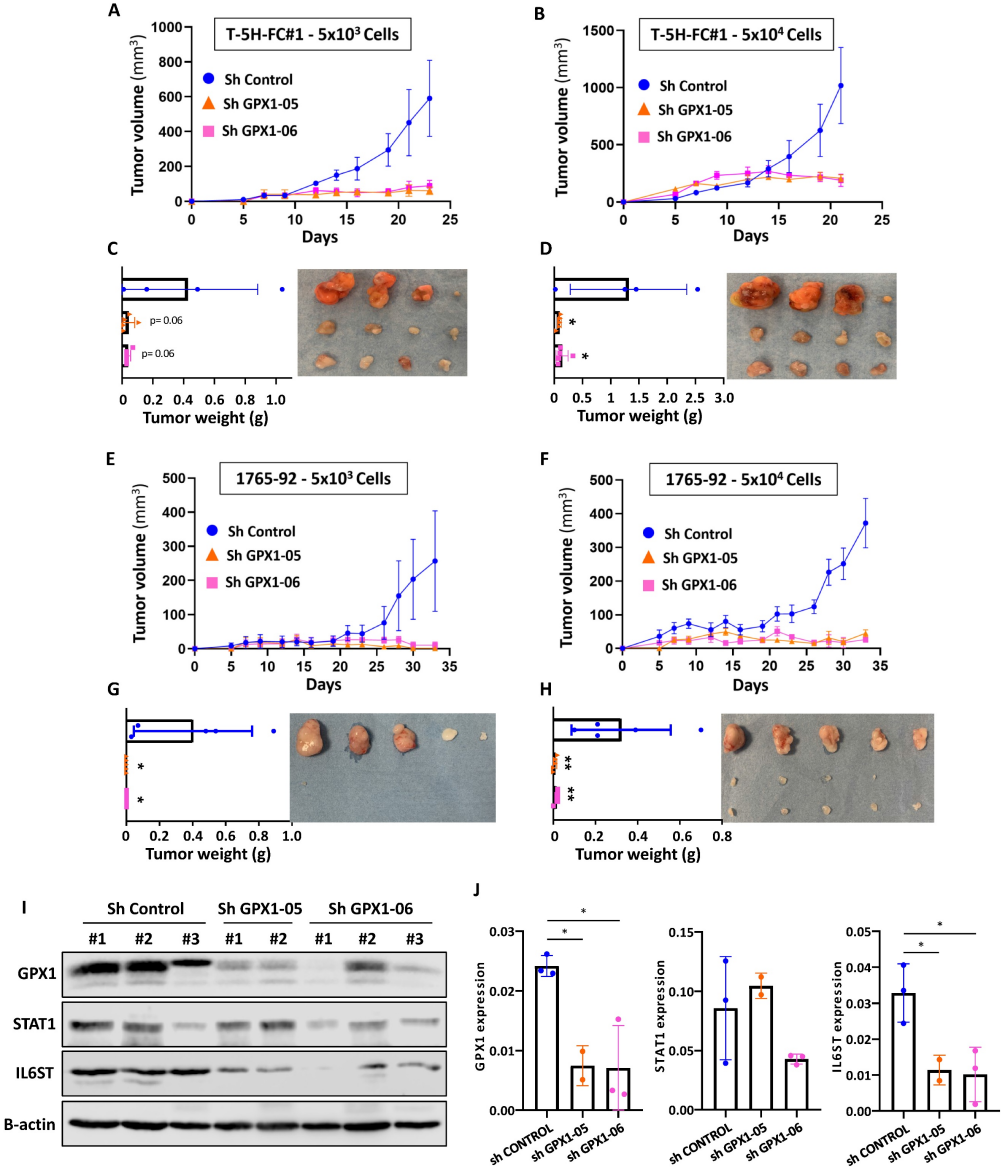
** Tumor formation ability of GPX1 depleted cells.** (A-B) Tumor growth kinetics observed after the inoculation into immunodeficient mice of 5 × 10^3^ (A) or 5 × 10^4^ (B) control (sh Control) and GPX1 depleted (sh GPX1-05 and sh GPX1-06) T-5H-FC#1 cells (n=4). (C-D) Tumor weights (left panels and images (right panels) of the tumors generated by T-5H-FC#1 cells extracted from the mice at the end of the experiment. (E-F) Tumor growth kinetics of 1765-92 cells assayed in the same conditions as in (A-B) (n=5). (G-H) weights (left panels) and images (right panels) of tumors generated by 1765-92 cells as in (C-D). (I) Western blotting analysis of STAT1 and IL6-ST in lysates obtained from tumors extracted from mice inoculated with control and GPX1-depleted T-5H-FC#1 cells (n=2). β-actin was used as a loading control. (J) Quantification of GPX1, STAT1 and IL6ST bands relative to those of β-actin. Due to the small size of the tumors generated by GPX1-depleted cells, we were only able to analyze two tumors in the sh GPX1-05 condition. Error bars represent the SEM (panels A, B, E and F) or SD (panels C, D, G, H and J) and asterisks indicate statistically significant differences between groups (*: p < 0.05; **: p < 0.01; one-way ANOVA).

Finally, we studied the effect of GPX1 depletion on the ability of sarcoma cells to initiate tumor growth *in vivo*. To better observe changes in tumorigenic capacity, we inoculated s.c. two dilutions with low cell density (50,000 and 5,000 cells) of T-5H-FC#1 (Figure 9A-D) or 1765-92 (Figure 9E-H) cell lines in immunodeficient mice. As expected, T-5H-FC#1 control cells (Sh Control) efficiently initiated tumor growth, being tumors generated after the inoculation of a higher number of cells three times larger after 23 days. GPX1 depletion with both shRNAs was able to block tumor growth, resulting in the formation of tumors with volumes six times smaller than those generated by control cells at both cell densities tested (Figure 9A-B). Confirming these findings, tumors generated by control cells weighed 11 times more than those generated by GPX1-depleted cells (Figure 9C-D).

The anti-tumor effect of depleting GPX1 was also observed in 1765-92 cells (Figure 9E-H). Tumors generated by 1765-92 control cells grow more slowly than those grown by T-5H-FC#1 control cells and in the case of mice inoculated with the lower cell density of GPX1-depleted cells, no tumor growth was even detected at the experimental endpoint (Figure 9E-G).

Western blotting analysis in tumors confirmed that the expression of GPX1 was severely repressed in T-5H-FC#1 cells carrying GPX1 shRNAs. Moreover, T-5H-FC#1-Sh GPX1-05 and -Sh GPX1-06 cells also showed reduced levels of proteins found downregulated in the proteomic profiling of GPX1-depleted cells, such as STAT1 and IL6ST. Thus, IL6ST was significantly downregulated in T-5H-FC#1 cells expressing both GPX1 Sh RNAs, and STAT1 showed reduced levels in T-5H-FC#1-Sh GPX1-06 cells (Figure 9 I-J).

These results confirm the pro-tumor effect of GPX1 expression in sarcoma cells and its relationship with a more aggressive phenotype.

## Discussion

CSCs are strongly associated with the evolution of tumors towards more aggressive behaviors. In the initial stages of tumor development, CSCs represent a small subset of cells with capabilities of self-renewal, differentiation and tumorigenic potential 48. However, during tumor progression to more aggressive phenotypes, it is suggested that the proportion of tumor cells presenting CSC-like features increases and is selected, so that, in advanced tumors virtually all tumor cells can disseminate and/or re-initiate tumor growth 49. As proof of this kind of tumor evolution, it has been shown that sarcoma cells increased both their stemness properties and tumorigenic potential after being sequentially transplanted and grown in mice 29, 50, 51. Therefore, cell line/xenograft line tandems, like the one formed by MSC-5H-FC, T-5H-FC#1 and T2-5H-FC#1, constitute valuable models to study CSCs subpopulations during tumor progression 29. By performing a proteomic analysis in bulk and CSC-enriched cultures of this model of sarcomagenesis, we have been able to identify targets associated with the acquisition of stemness properties in the different steps of evolution toward more aggressive phenotypes in our model.

Experimental oncogenic transformation, such as that induced in MSC-5H-FC cells, has been shown to cause a significant increase in ROS and, therefore, a deregulation of the signaling that processes these species 52, 53. In addition, the increase in oxidative stress burden during the initial stages of tumor development may lead in some tumor types to a dependence of tumor cells on antioxidant defense mechanisms 9. In our proteomic analysis, we found that the antioxidant factor GPX1 was upregulated in CSC-enriched tumorspheres in all cell types of the sarcomagenesis model. By attenuating the accumulation of ROS, GPX1 plays relevant roles in controlling the physiological homeostasis of many biological systems 54 and is required for self-renewal of murine embryonic stem cells 55. In concordance with the dual role that the control of ROS may play in cancer, GPX1 is also reported to act as a tumor-suppressor or promoter factor according to the tumor type and/or tumor stage 13. As in the group of tumor types where GPX1 plays pro-tumorigenic roles, here we show that the expression of GPX1 correlates with more aggressive phenotypes and a worse prognosis in sarcoma patients, and favors proliferation, migration, sphere-forming ability and tumorigenic potential in sarcoma cells. In a similar way, it has been reported that colon cancer cells depleted of GPX2 showed a reduced capacity to grow as colonospheres and were less tumorigenic *in vivo* 56. Although an increase in the expression of pluripotency factors was also observed, the pro-tumor phenotype achieved after the depletion of GPX2 was associated with a reduction of the self-renewal, differentiation, and metastatic potential of cancer cells, thus suggesting that the knockdown of this anti-oxidant factor resulted in the generation of a non-functional and less aggressive CSC population 56. Moreover, essential roles in maintaining aggressive phenotypes and stemness were also described for GPX2 in gastric cancer 57 and GPX8 in breast cancer 58.

Besides the tumor-type dependent effects of GPXs, the timing of tumor progression could also influence the effects of these antioxidant factors in cancer. Specifically, there is a certain consensus that GPX1 may have a protective role in cancer initiation, mainly through the prevention of ROS-mediated DNA damage 14. In this regard, our somehow contradictory finding regarding the increased colony-forming ability of GPX1-depleted cells may reflect the role of this enzyme in restraining a property, such as the clonogenicity potential, related to the tumor initiation process. It is also worth noting that GPX1 depletion induced the upregulation of factors such as UCHL1, which has been implicated in the stemness phenotype in other tumor types 41, 42. This observation raises the possibility that UCHL1 might play a role in enhancing colony-forming unit (CFU) formation in sarcoma cells. In addition, it is plausible to speculate that CFU cultures select for a subpopulation of cells in which GPX1 regulates stem-related properties differently than in monolayer and tumorsphere cultures.

Proteomic analysis revealed that relevant interconnected inflammatory pathways, such as those mediated by IFNα, IFNγ, IL6-JAK-STAT3, and TNFα/NFκB, were downregulated in GPX1-deficient cells. This pattern of deregulation was also observed after the knockdown of GPX8 in breast cancer 58. That study shows that this GPX factor plays an essential role in maintaining aggressiveness (EMT signaling and tumor growth potential) and stemness (mammosphere-forming ability) through the autocrine activation of IL6/JAK/STAT3 signaling. Similarly, the depletion of GPX1 in our model resulted in the significant downregulation of key factors of the IL6-JAK-STAT signaling such as the co-receptor IL6-ST whose activation is imperative for transducing IL6-induced signaling.

STAT3 signaling is also a relevant pro-stemness cue that may favor the emergence of CSCs and tumor growth in response to ROS in osteosarcoma 59, 60. Therefore, the inhibition of STAT3 signaling may contribute to the repression of pro-stemness features in our MLS model upon GPX1 depletion.

Interestingly, ROS accumulation during MSC transformation has been reported to correlate with transcriptional downregulation of NRF2 and other downstream antioxidant genes 52. However, GPX1, whose expression in mesenchymal cell types may be regulated by NRF2-alternative mechanisms such as NFkB and AP-1 61, was not altered in transformed MSCs. Taken together, it can be speculated that transformed MSCs become dependent on the anti-redox activity of GPX1 to cope with excessive oxidative stress and therefore targeting this factor may reduce the fitness and aggressiveness of sarcoma cells.

In sum, our results show that GPX1 is upregulated in CSC subpopulations of MLS cells, where it plays a relevant role in maintaining aggressiveness-related features. The inhibition of this antioxidant factor may revert the phenotype of CSCs to a more primitive and less aggressive phenotype, also offering a potential vulnerability to be explored for the treatment of sarcomas.

## Supplementary Material

Supplementary figures and tables.

## Abbreviations

CSCs: cancer stem cells
CFU: colony formation unit
DEPs: differentially expressed proteins
FDR: false discovery rate
GPX: glutathione peroxidase
GSEA: gene set enrichment analysis
MLS: myxoid liposarcoma
MSCs: mesenchymal stromal/stem cells
PCA: principal component analysis
ROS: reactive oxygen species
