# Supplementary material for: GPX1 expression promotes stemness and aggressiveness in myxoid liposarcomas
Source: Int J Biol Sci. 2025 Aug 30;21(13):5609–27. doi: 10.7150/ijbs.105217 (PMC12509692; doi:10.7150/ijbs.105217)
Supplement: Supplementary file 1 — Supplementary figures and tables. [file ijbsv21p5609s1.pdf]

Figure S1

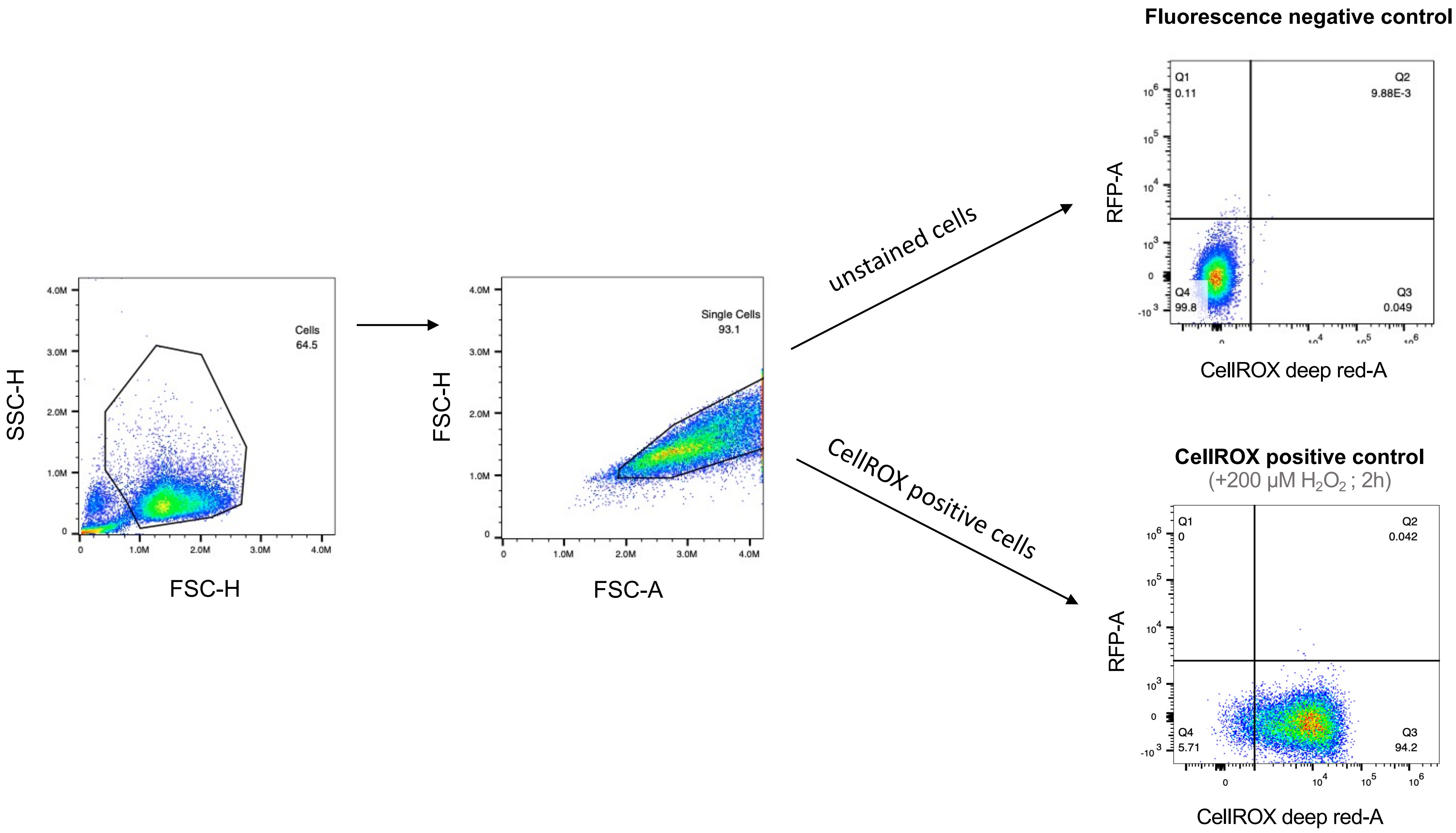

Figure S1. Analysis of ROS by flow cytometry using CellROX assay. Gating strategy used to analyzed the amount of ROS in 1765-92 cells. Fluorescence negative controls and positive controls (1765-92 cells treated with 200 µM H2O2 for 2 hours) were used to stablish the gate for CellROx positive cells.

Figure S2

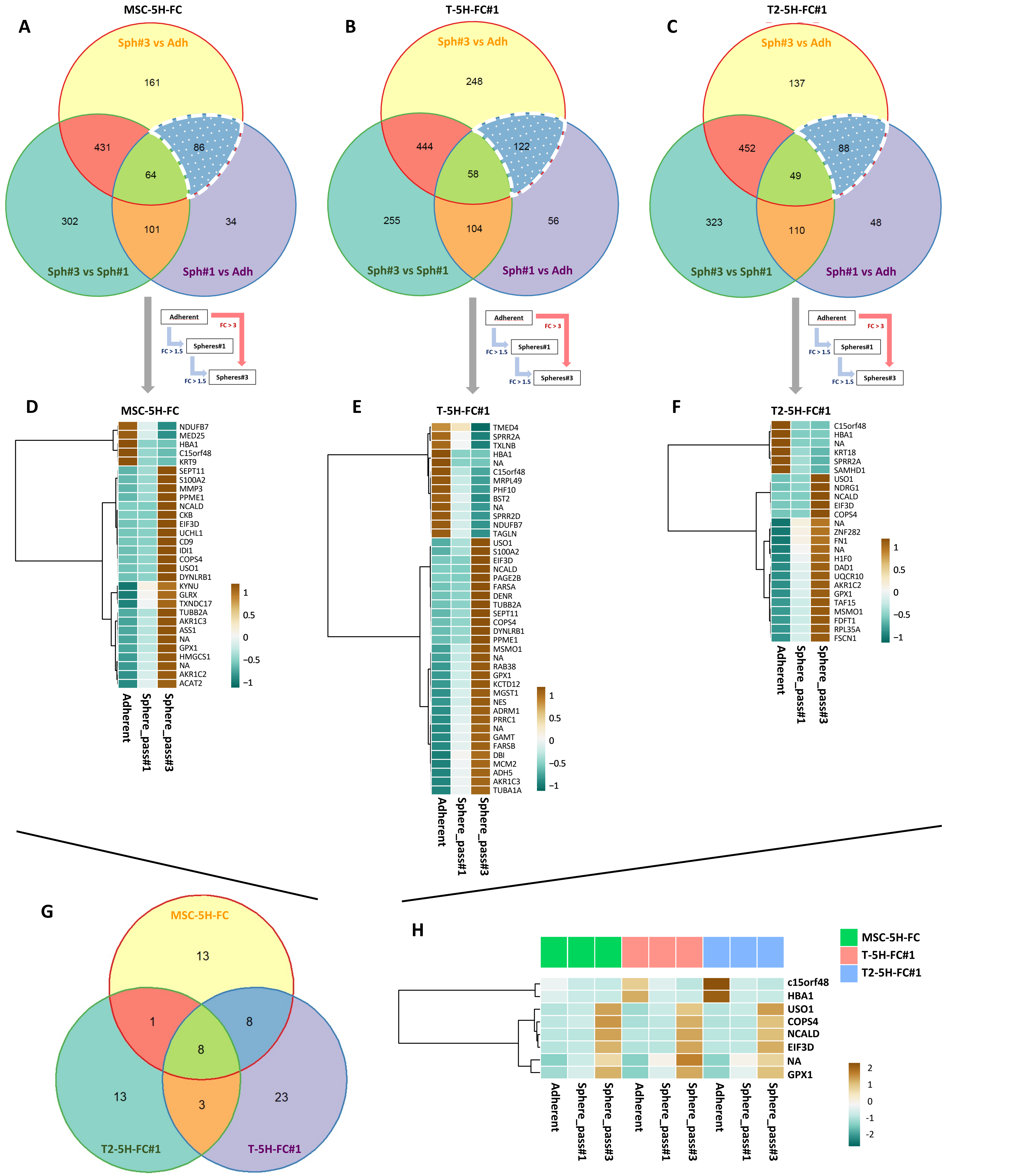

Figure S2. Proteomic analysis of CSC-enriched subpopulations in MSC-5H-FC, T-5H-FC#1 and T2-5H-FC#1. (A-C) Top panels: Venn diagram displaying the intersections between selected proteins (log2 (FC) ≤-0.5 or ≥0.5) in Sph1 vs Adh, Sph3 vs Adh and Sph3 vs Sph1 comparisons in MSC-5H-FC (A), T-5H-FC#1 (B) and T3-5H-FC#1 (C) cells. Bottom panels: Scheme with criteria for the selection of proteins of interest (as in Figure 1F). (D-G) Heat map showing those proteins selected according the criteria described in Figure 1F in MSC-5H-FC (D), T-5H-FC#1 (E) and T3-5H-FC#1 (F) cells. (G) Venn diagram displaying the intersections between proteins selected in MSC-5H-FC, T-5H-FC#1 and T3-5H-FC#1 cells (as in Figure 1G). (H) Heat map showing commonly selected targets across all cell lines (as in Figure 1H). NA: not identified peptide.

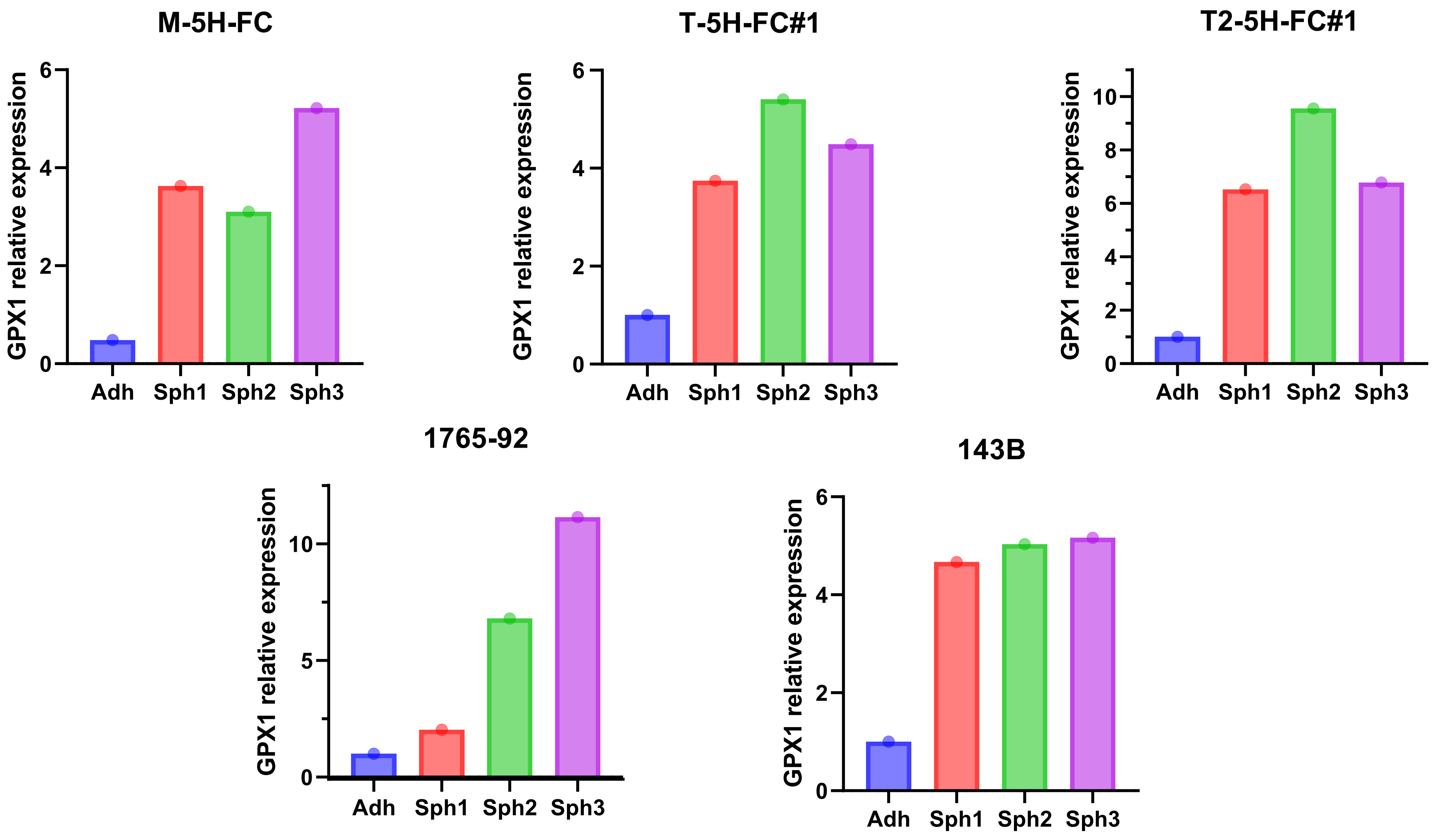

Figure S3. Quantification of GPX1 levels in the Western blotting analyses shown in Figure 1K. Quantification of GPX1 levels relative to the corresponding β-actin levels in adherent (Adh) and tumorsphere passages (Sph1-3) of the indicated cell lines. Data are presented as fold change relative to the Adh condition for each cell line.

Figure S4

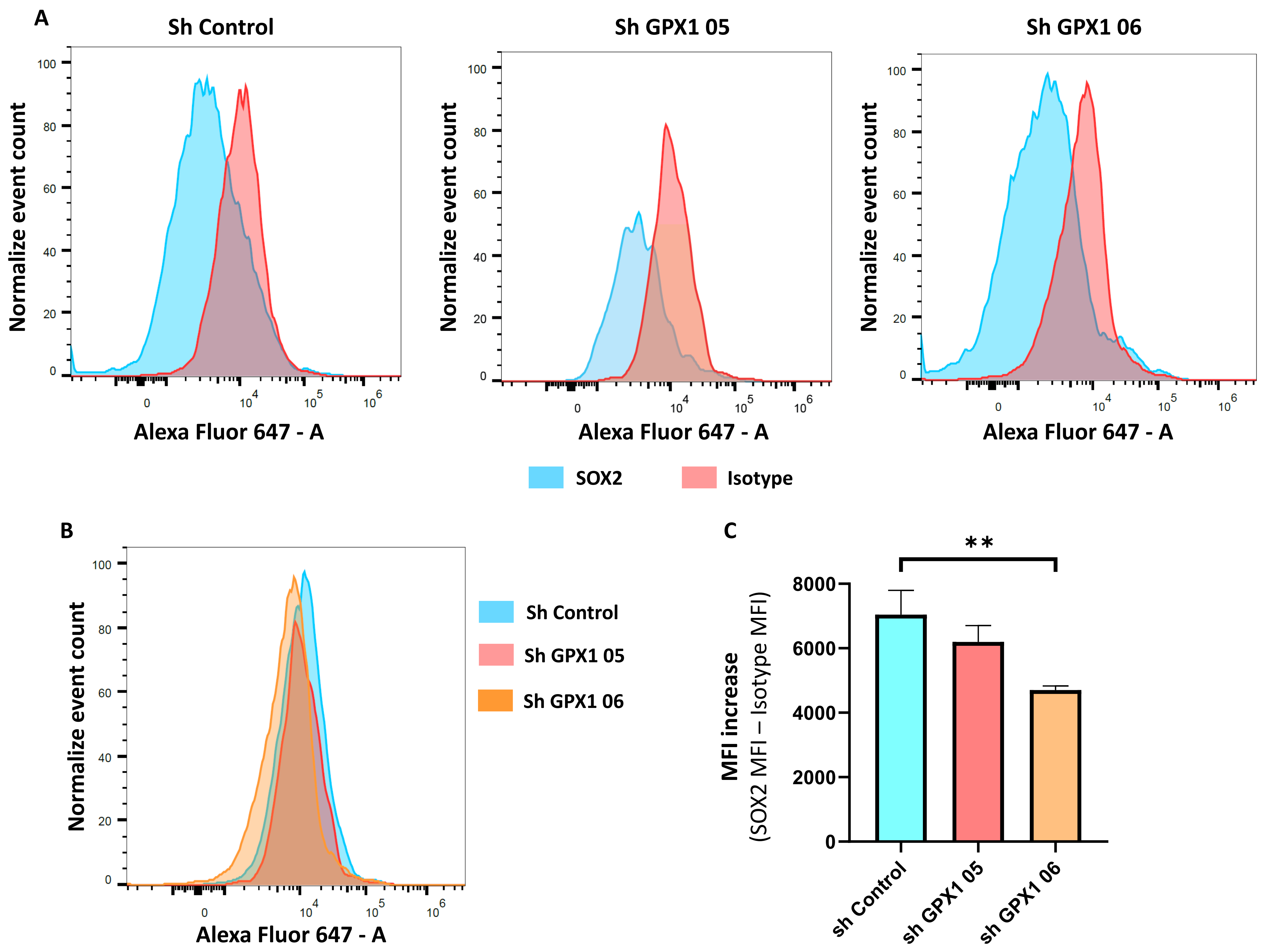

Figure S4. Flow cytometry analysis of SOX2 expression levels in control and GPX1-depleted T5H-FC#1 cells. (A) Representative histograms of the indicated cell types incubated with an anti-SOX2 antibody or the corresponding isotype. (B) Representative overlay of histograms of control and GPX-1-depleted cells incubated with anti-SOX2. (C) Mean and standard deviation of the difference in Median Fluorescence Intensity (MFI) between cells incubated with anti-SOX2 and those incubated with isotype control across three independent experiments, showing reduced SOX2 expression in shGPX1-05 ($p = 0.200$) and shGPX1-06 cells ($p = 0.004$) compared to shControl cells. Asterisks indicate statistically significant differences (**: $p < 0.01$; two-way ANOVA).

Figure S5

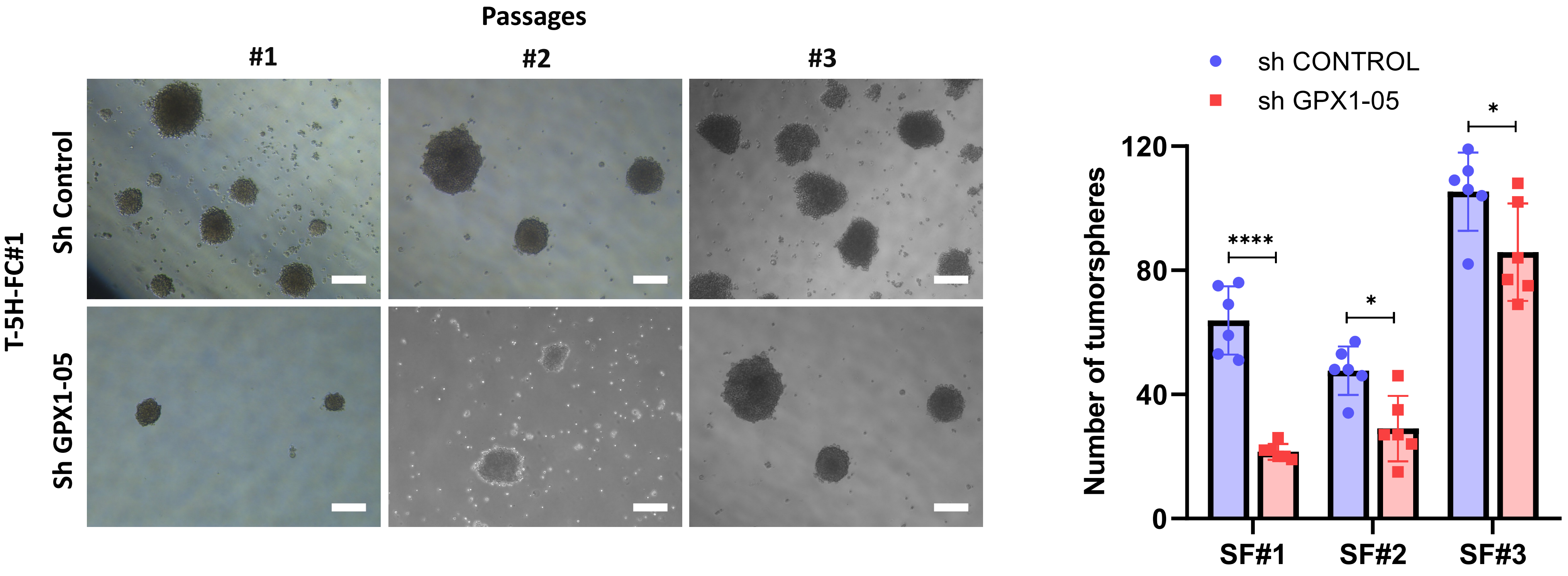

Figure S5. Tumorsphere-forming ability of control and GPX1-depleted cells after serial passages. (A) Representative images of tumorsphere cultures of Sh Control and Sh GPX1-05 T-5H-FC#1 cells in passages #1, #2 and #3 (scale bars = 200 µm). (B) Quantification of the number of tumorspheres at each passage. Asterisks indicate statistically significant differences (*: $p < 0.05$; ****: $p < 0.0001$; two-way ANOVA).

Figure S6

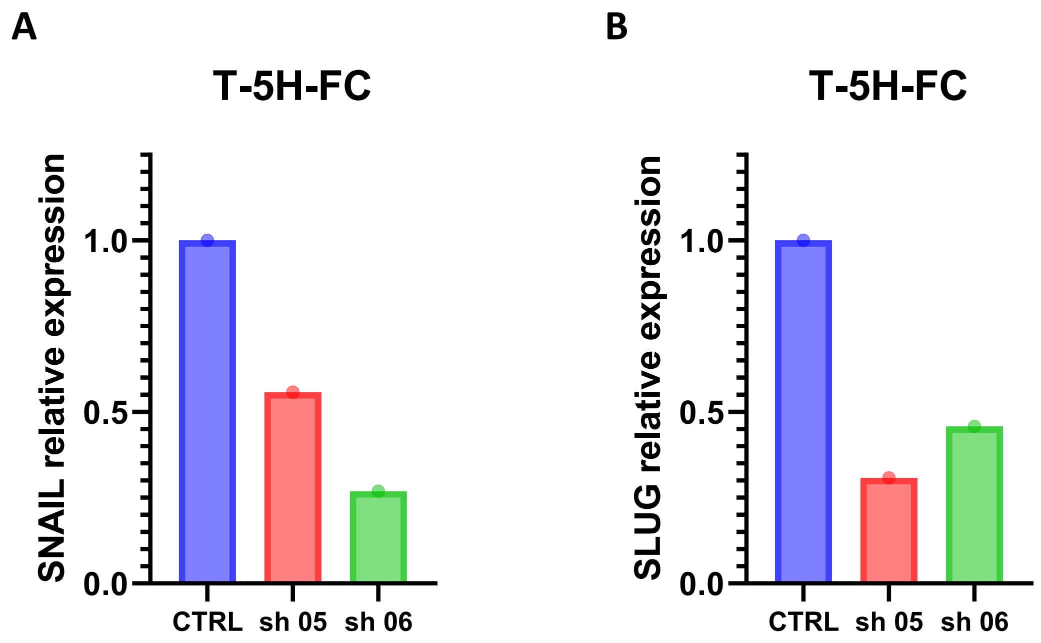

Figure S6. Quantification of the Western blotting analyses shown in Figure 4C. Quantification of SNAIL (A) and SLUG (B) levels relative to the corresponding β-actin levels in control and GPX1 depleted cells (sh 05 and sh 06) T-5H-FC#1 cells. Data are presented as fold change relative to the control condition.

Figure S7

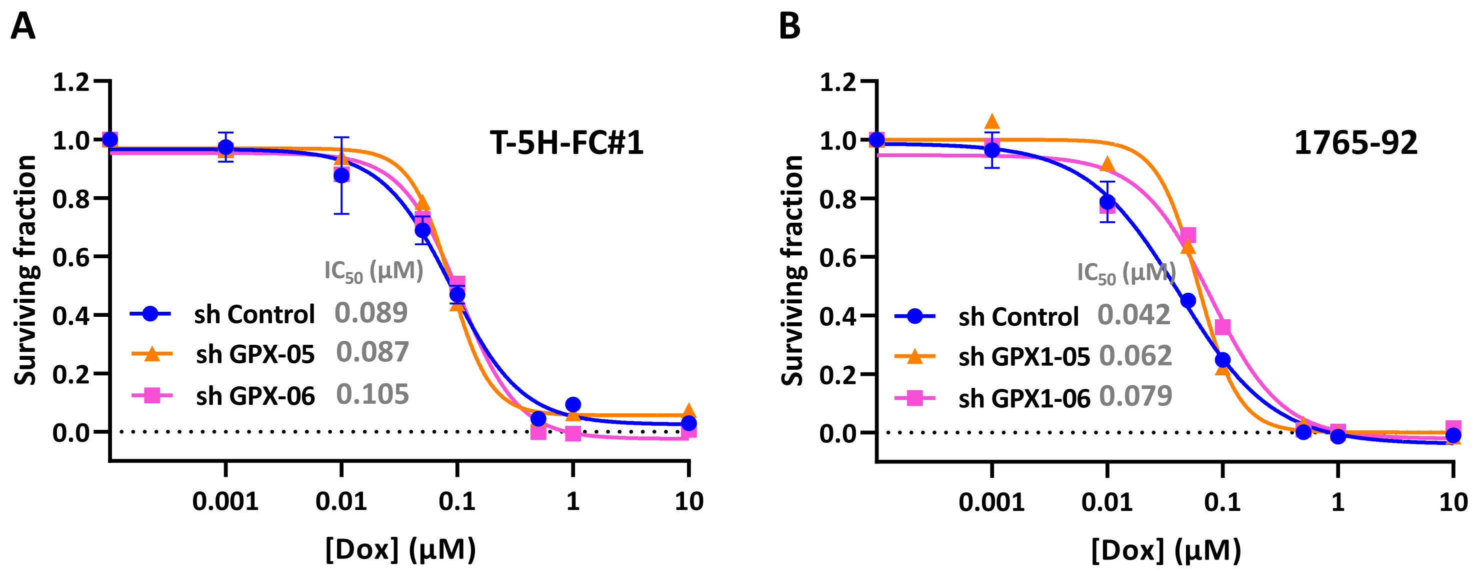

Figure S7. Effect of Doxorubicin on cell viability. Cell viability (WST-1 assays) was measured after the treatment of control and GPX1 depleted T-5H-FC#1 (A) and 1765-92 (B) cells with increasing concentrations of doxorubicin for 72 h.

Figure S8

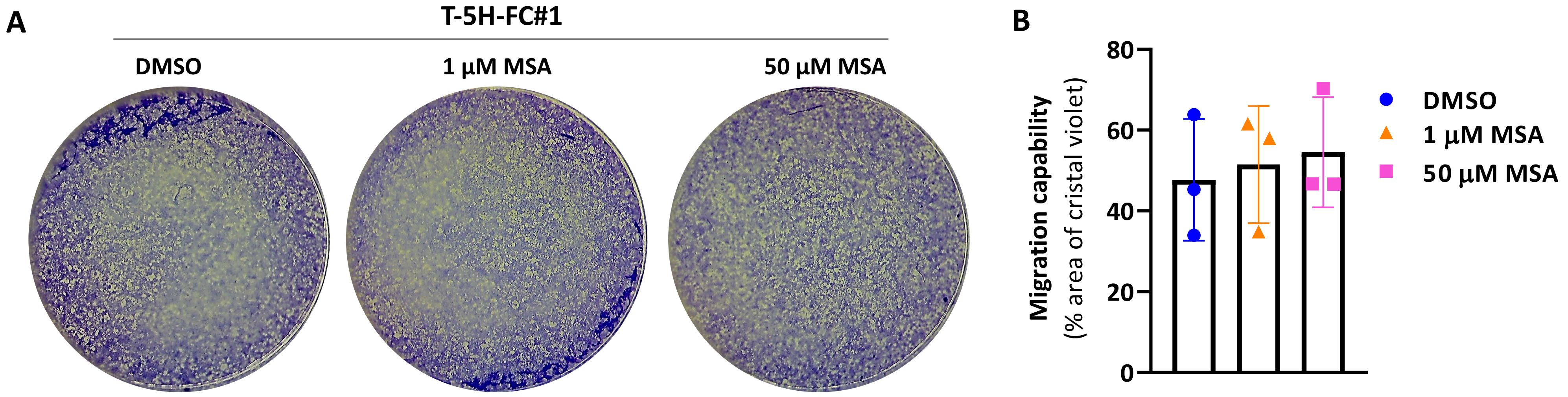

Figure S8. Effect of MSA on the migration capability of sarcoma cells. (E-F) Transwell migration assay of T-5H-FC#1 cells treated with DMSO (vehicle), 1 µM or 50 µM MSA. Representative images (E) and quantification of the surface occupied by migrated cells (F) are shown.

Figure S9

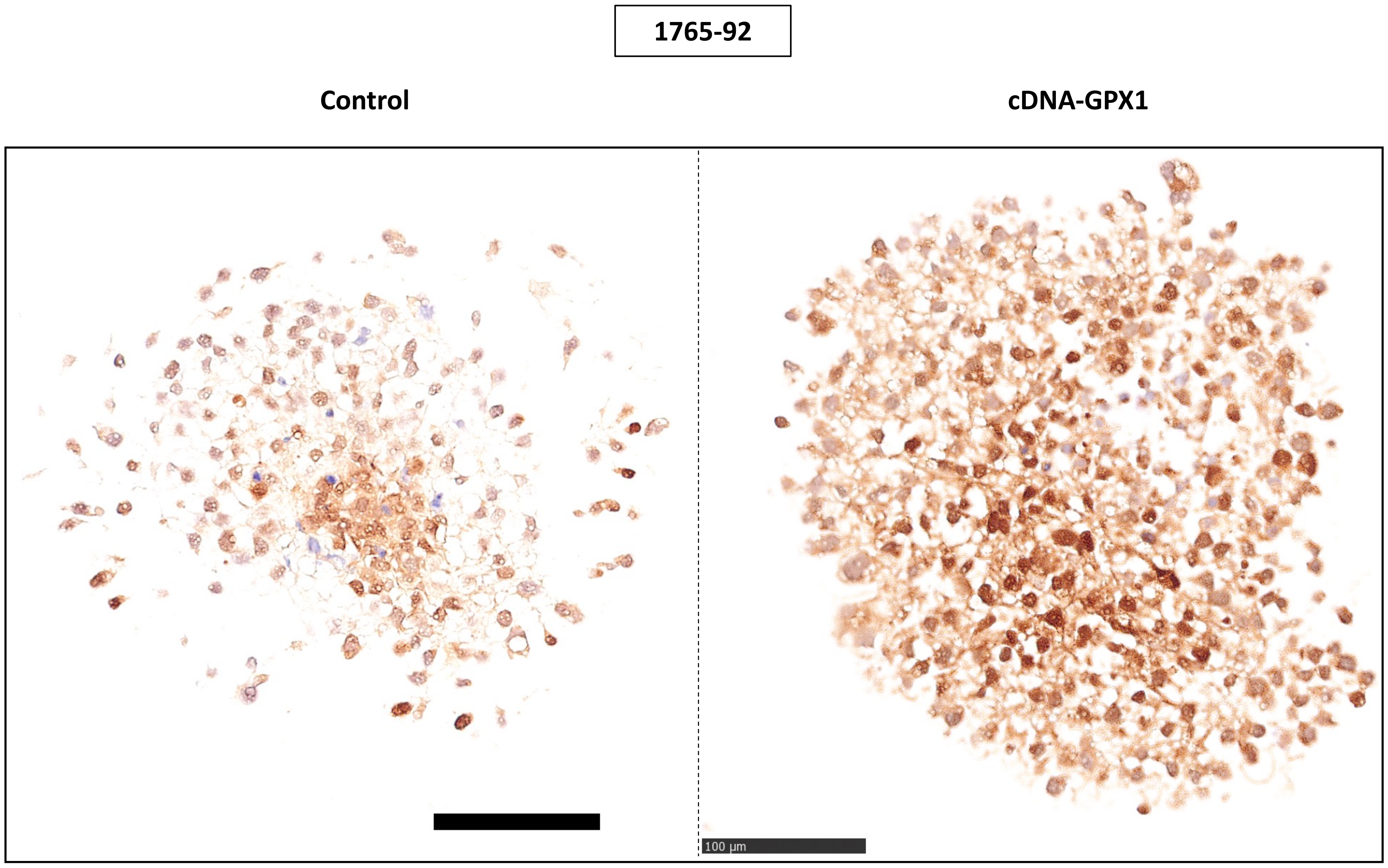

Figure S9. Immunostaining of GPX1 in tumorspheres formed by control- and cDNA-GPX1-1765-92 cells confirmed a higher level of expession of this anti-oxidant factor in GPX1-overexpressing spheres. Scale bars = 100 µm.

Supplementary Table S1. Differentially expressed proteins in MSC-5H-FC cells which expression: 1) increased or decreased constantly (Log2FC ≤-0.5 or ≥ 0.5) throughout all passages (Sph3 ≥ Sph1 ≥ Adh or Sph3 ≤ Sph1 ≤ Adh); or 2) showed at least a three-fold change (log2FC ≤-1.5 or ≥1.5) in expression between third-generation spheres and adherent cultures.

	X1.adherent_1.MSC.5H.FC1	X2.1st.pass_1.MSC.5H.FC1	X3.3rd.pass_1.MSC.5H.FC1	Los2 fc_M_sf1_vs_adh	log2fc_M_sf3_vs_sf1	log2fc_M_sf3_vs_adh	SYMBOL	CHANGE
A0A024R5U4	23.7875	7.675	5.8875	-1.631965001	-0.382511596	-2.014476597	C15orf48	DOWN
A0A024RAM2	58.7375	139.225	213.3	1.245064525	0.615465673	1.860530198	GLRX	UP
A0A024RDG1	27.75	31.375	84.3125	0.177127688	1.426131079	1.603258767	USO1	UP
B2RB70	0.4	0.4	19.1125	0	5.578372691	5.578372691	NCALD	UP
D1MGQ2	15.2625	4.075	3.4	-1.905119331	-0.261265313	-2.166384644	HBA1	DOWN
O15371	26.2375	29.025	84.6875	0.145665806	1.544852974	1.69051878	EIF3D	UP
P00966	22.2	75	264.2375	1.756330919	1.816872724	3.573203644	ASS1	UP
P08254	19.125	26.25	73.8625	0.456857675	1.492524671	1.949382346	MMP3	UP
P09936	47.85	57.45	280.0625	0.263787968	2.285370023	2.549157992	UCHL1	UP
P17568	29.05	19.5	13.325	-0.57506404	-0.549338591	-1.124402631	NDUFB7	DOWN
P21926	51.7375	56.325	162.65	0.122565065	1.529923509	1.652488574	CD9	UP
P29034	9.125	23.925	139.275	1.390622461	2.541345486	3.931967947	S100A2	UP
P35527	197.3125	63.6	50.9625	-1.633383685	-0.319590714	-1.952974399	KRT9	DOWN
P42330	30.475	51.625	115.1875	0.760443656	1.157842384	1.91828604	AKR1C3	UP
P52895	17.3375	36.25	70.1625	1.064085113	0.952719159	2.016804272	AKR1C2	UP
Q01581	43.0375	70.575	138.2375	0.713562948	0.969919903	1.683482851	HMGCS1	UP
Q13885	32.275	46.225	103.775	0.518256224	1.166713707	1.684969931	TUBB2A	UP
Q13907	45.25	52.075	130.425	0.202673142	1.324557594	1.527230736	IDI1	UP
Q16719	12.1875	36.375	54.325	1.577545029	0.578669021	2.156214051	KYNU	UP
Q53H26	20.75	42.425	93.4625	1.031803323	1.139472968	2.171276291	NA	UP
Q59GM9	29.45	56.325	145.6	0.935507774	1.370163042	2.305670816	NA	UP
Q71SY5	32.3375	18.275	10.3625	-0.823336743	-0.818499304	-1.641836047	MED25	DOWN
Q7L4Q3	31.0625	48.15	104.7875	0.632361851	1.121858926	1.754220778	GPX1	UP
Q9BRA2	68.45	126.5	190.6375	0.886014938	0.591694552	1.477709491	TXNDC17	UP
Q9BT78	31.5	36.875	107.125	0.227291221	1.53858025	1.765871471	COPS4	UP
Q9BWD1	61.4625	92.025	136.0375	0.582319395	0.563906649	1.146226043	ACAT2	UP
Q9NP97	27.7	31.55	87.4625	0.187754029	1.471024581	1.65877861	DYNLRB1	UP
Q9NVA2	26.8875	36.425	92.65	0.437993353	1.346862004	1.784855357	SEPT11	UP
Q9Y570	33.8875	42.35	102.4375	0.321608761	1.274310075	1.595918836	PPME1	UP
V9HWH2	12.8	12.8	120.5125	0	3.234967081	3.234967081	CKB	UP

Supplementary Table S2. Differentially expressed proteins in T-5H-FC#1 cells which expression: 1) increased or decreased constantly (Log2FC ≤-0.5 or ≥ 0.5) throughout all passages (Sph3 ≥ Sph1 ≥ Adh or Sph3 ≤ Sph1 ≤ Adh); or 2) showed at least a three-fold change (log2FC ≤-1.5 or ≥1.5) in expression between third-generation spheres and adherent cultures.

	X1.adherent_2.T.5H.FC1	X2.1st.pass_2.T.5H.FC1	X3.3rd.pass_2.T.5H.FC1	Los2 fc_T_sf1_vs_adh	log2fc_T_sf3_vs_sf1	log2fc_T_sf3_vs_adh	SYMBOL	CHANGE
A0A024R191	24.925	36.85	63.4625	0.564071115	0.784239736	1.34831085	RAB38	UP
A0A024R5U4	63.525	17.225	3.4	-1.882820487	-2.340897331	-4.223717819	C15orf48	DOWN
A0A024RBR3	28.7	32.05	82.55	0.15927362	1.364943858	1.524217478	DENR	UP
A0A024RDG1	22.925	32.425	75.125	0.500184841	1.212186511	1.712371352	USO1	UP
B2RB70	0.4	0.4	18.75	0	5.550746785	5.550746785	NCALD	UP
D1MGQ2	90.775	7.35	3.7	-3.62647887	-0.990218979	-4.616697849	HBA1	DOWN
K7WVJ5	74.3	44.9	31.1625	-0.726646766	-0.526904467	-1.253551233	NA	DOWN
O15371	25.3	26.75	88.4375	0.080401507	1.725119351	1.805520858	EIF3D	UP
P07108	50.175	93.85	134.6625	0.903388033	0.520919504	1.424307537	DBI	UP
P10620	75.475	112.225	176.75	0.572323339	0.655316119	1.227639458	MGST1	UP
P17568	42.775	22.4	11.9875	-0.933269122	-0.901967917	-1.835237039	NDUFB7	DOWN
P22532	281.25	109.1	28.0125	-1.366201995	-1.961508453	-3.327710447	SPRR2D	DOWN
P29034	31.825	43.775	102.125	0.459946665	1.222156995	1.682103659	S100A2	UP
P35326	87.525	55.9	25.075	-0.646846874	-1.156598582	-1.803445456	SPRR2A	DOWN
P42330	26.9	53.2	84.475	0.983820073	0.6670982	1.650918273	AKR1C3	UP
P48681	39.225	67.225	123.8125	0.777224403	0.88108722	1.658311623	NES	UP
P49736	55.5	91.25	130.1875	0.717336787	0.51269447	1.230031258	MCM2	UP
Q10589	49.525	30.25	17.5375	-0.711221833	-0.786492039	-1.497713871	BST2	DOWN
Q13405	44.225	21.3	12.45	-1.054008712	-0.774707688	-1.828716401	MRPL49	DOWN
Q13885	23.1	27.075	86.9625	0.229068486	1.683432078	1.912500564	TUBB2A	UP
Q14353	27.9	43.025	63.9375	0.62491007	0.571487143	1.196397213	GAMT	UP
Q15800	40.7	62.2	122.0875	0.611885786	0.972929011	1.584814797	MSMO1	UP
Q16186	60.075	90.425	137.9375	0.58995695	0.609221127	1.199178076	ADRM1	UP
Q53H26	20.575	68.1	129.6375	1.726762368	0.928756401	2.655518769	NA	UP
Q59GM9	24.8	51.675	126.0875	1.059126363	1.286886869	2.346013232	NA	UP
Q5JRK9	31.9	31.9	96.8875	0	1.602754123	1.602754123	PAGE2B	UP
Q5U0D2	139.9	88.575	59.4375	-0.659424497	-0.575526124	-1.234950621	TAGLN	DOWN
Q6IBR2	35.175	38.9	101.4	0.145219732	1.382215592	1.527435324	FARSA	UP
Q6IRT1	62.575	95.125	134.8125	0.604238163	0.503057818	1.10729598	ADH5	UP
Q71U36	33.15	72.275	118.425	1.124487833	0.712405063	1.836892896	TUBA1A	UP
Q7L4Q3	24.5	50.025	107.2625	1.029867513	1.100424617	2.13029213	GPX1	UP
Q7Z7H5	34.675	28.4	10.2125	-0.288004953	-1.475554851	-1.763559804	TMED4	DOWN
Q8N3L3	121.675	80.425	40.4375	-0.597316839	-0.991950224	-1.589267062	TXLNB	DOWN
Q8WUB8	50.7	27.9	18.5875	-0.861720625	-0.58593238	-1.447653005	PHF10	DOWN
Q96CX2	40.3	57.025	94.675	0.500814703	0.731388975	1.232203677	KCTD12	UP
Q96M27	66.55	98.125	146.1	0.560182083	0.574263524	1.134445607	PRRC1	UP
Q9BT78	25.75	31.6	99.65	0.295352126	1.656945247	1.952297373	COPS4	UP
Q9NP97	20.05	24.8	69.1625	0.306737884	1.479649898	1.786387782	DYNLRB1	UP
Q9NSD9	61.2	91.8	134.6375	0.584962501	0.552514235	1.137476735	FARSB	UP
Q9NVA2	22.05	29.25	80.5375	0.407657969	1.461224065	1.868882034	SEPT11	UP
Q9NZ23	262.8	31.325	20.575	-3.068578861	-0.606422079	-3.67500094	NA	DOWN
Q9Y570	25.025	29.675	74.9375	0.245877961	1.336439819	1.582317779	PPME1	UP

Supplementary Table S3. Differentially expressed proteins in T2-5H-FC#1 cells which expression: 1) increased or decreased constantly (Log2FC ≤-0.5 or ≥ 0.5) throughout all passages (Sph3 ≥ Sph1 ≥ Adh or Sph3 ≤ Sph1 ≤ Adh); or 2) showed at least a three-fold change (log2FC ≤-1.5 or ≥1.5) in expression between third-generation spheres and adherent cultures.

	X1.adherent_3.T2.5H.FC1	X2.1st.pass_3.T2.5H.FC1	X3.3rd.pass_3.T2.5H.FC1	Los2 fc_T2_sf1_vs_adh	log2fc_T2_sf3_vs_sf1	log2fc_T2_sf3_vs_adh	SYMBOL	CHANGE
A0A024R462	24.175	118.325	208.725	2.291167127	0.818848487	3.110015615	NA	UP
A0A024R5U4	112.775	10.025	4.1625	-3.491773144	-1.268080059	-4.759853204	C15orf48	DOWN
A0A024RDG1	27.95	31.95	85.5625	0.192967648	1.421162705	1.614130353	USO1	UP
A0A090N8Y3	38.8	130.75	198.7625	1.752682389	0.604234647	2.356917036	ZNF282	UP
B2RB70	0.4	0.4	16.7875	0	5.391243589	5.391243589	NCALD	UP
D1MGQ2	127	4.975	3.4125	-4.673988161	-0.54386748	-5.217855641	HBA1	DOWN
O15371	27.175	28.575	86.175	0.072473463	1.592515895	1.664989358	EIF3D	UP
P02751	39.075	112.75	167.3	1.528809655	0.569310012	2.098119667	FN1	UP
P05783	166.425	56.2	40.7375	-1.566230133	-0.464212683	-2.030442816	KRT18	DOWN
P07305	72.3	122.8	182.3625	0.764243008	0.570498532	1.33474154	H1F0	UP
P18077	86.95	130.075	219.825	0.581085775	0.757011761	1.338097536	RPL35A	UP
P35326	99.2	36.525	25.05	-1.441455848	-0.54407367	-1.985529517	SPRR2A	DOWN
P52895	24.75	35.275	52.8625	0.511217558	0.583598572	1.09481613	AKR1C2	UP
P61803	66.65	98.95	147.025	0.570094832	0.571289878	1.14138471	DAD1	UP
Q15800	42.075	72.525	142	0.785515119	0.969340634	1.754855753	MSMO1	UP
Q16658	69.775	101.8	161.4375	0.544955437	0.665238177	1.210193614	FSCN1	UP
Q53H26	22.4	67.3	97.2125	1.587107773	0.530535329	2.117643101	NA	UP
Q6IAX1	44.2	64.75	112.25	0.550833823	0.793763347	1.34459717	FDFT1	UP
Q7L4Q3	27.2	54.25	99.9375	0.996016486	0.881402991	1.877419477	GPX1	UP
Q92597	62.475	71.9	208.7875	0.202712774	1.537971665	1.74068444	NDRG1	UP
Q92804	62.65	93.775	163.375	0.581888848	0.800911973	1.382800821	TAF15	UP
Q9BT78	27.95	30.65	93.6375	0.133038791	1.611199343	1.744238134	COPS4	UP
Q9NZ23	244.85	25.375	19.6625	-3.270418469	-0.367961057	-3.638379525	NA	DOWN
Q9UDW1	65.725	94.55	142.025	0.524635221	0.58699554	1.111630761	UQCR10	UP
Q9Y3Z3	50.4	18.4	12.6	-1.453717967	-0.546282033	-2	SAMHD1	DOWN

Supplementary Table S4. List of common proteins in that MSC-5H-FC, T-5H-FC#1 and T2-5H-FC#1 cells which expression: 1) increased or decreased constantly (Log2FC ≤-0.5 or ≥ 0.5) throughout all passages (Sph3 ≥ Sph1 ≥ Adh or Sph3 ≤ Sph1 ≤ Adh); or 2) showed at least a three-fold change (log2FC ≤-1.5 or ≥1.5) in expression between third-generation spheres and adherent cultures.

	X1.adherent_1.MSC.5H.FC1	X2.1st.pass_1.MSC.5H.FC1	X3.3rd.pass_1.MSC.5H.FC1	Log2 fc_M_sf1_vs_adh	log2fc_M_sf3_vs_sf1	log2fc_M_sf3_vs_adh	X1.adherent_2.T.5H.FC1	X2.1st.pass_2.T.5H.FC1	X3.3rd.pass_2.T.5H.FC1	Log2 fc_T_sf1_vs_adh	log2fc_T_sf3_vs_sf1	log2fc_T_sf3_vs_adh	X1.adherent_3.T2.5H.FC1	X2.1st.pass_3.T2.5H.FC1	X3.3rd.pass_3.T2.5H.FC1	Log2 fc_T2_sf1_vs_adh	log2fc_T2_sf3_vs_sf1	log2fc_T2_sf3_vs_adh	SYMBOL	CHANGE
A0A024R5U4	23.7875	7.675	5.8875	-1.631965001	-0.382511596	-2.014476597	63.525	17.225	3.4	-1.882820487	-2.340897331	-4.223717819	112.775	10.025	4.1625	-3.491773144	-1.268080059	-4.759853204	C15orf48	DOWN
A0A024RDG1	27.75	31.375	84.3125	0.177127688	1.426131079	1.603258767	22.925	32.425	75.125	0.500184841	1.212186511	1.712371352	27.95	31.95	85.5625	0.192967648	1.421162705	1.614130353	USO1	UP
B2RB70	0.4	0.4	19.1125	0	5.578372691	5.578372691	0.4	0.4	18.75	0	5.550746785	5.550746785	0.4	0.4	16.7875	0	5.391243589	5.391243589	NCALD	UP
D1MGQ2	15.2625	4.075	3.4	-1.905119331	-0.261265313	-2.166384644	90.775	7.35	3.7	-3.62647887	-0.990218979	-4.616697849	127	4.975	3.4125	-4.673988161	-0.54386748	-5.217855641	HBA1	DOWN
O15371	26.2375	29.025	84.6875	0.145665806	1.544852974	1.69051878	25.3	26.75	88.4375	0.080401507	1.725119351	1.805520858	27.175	28.575	86.175	0.072473463	1.592515895	1.664989358	EIF3D	UP
Q53H26	20.75	42.425	93.4625	1.031803323	1.139472968	2.171276291	20.575	68.1	129.6375	1.726762368	0.928756401	2.655518769	22.4	67.3	97.2125	1.587107773	0.530535329	2.117643101	NA	UP
Q7L4Q3	31.0625	48.15	104.7875	0.632361851	1.121858926	1.754220778	24.5	50.025	107.2625	1.029867513	1.100424617	2.13029213	27.2	54.25	99.9375	0.996016486	0.881402991	1.877419477	GPX1	UP
Q9BT78	31.5	36.875	107.125	0.227291221	1.53858025	1.765871471	25.75	31.6	99.65	0.295352126	1.656945247	1.952297373	27.95	30.65	93.6375	0.133038791	1.611199343	1.744238134	COPS4	UP

Supplementary Table S5. List of differentially expressed proteins in T5H-FC#1-sh GPX1-05 vs. T5H-FC#1- sh Control conditions.

(Cont.)

Column1	logFC	AveExpr	t	P.Value	adj.P.Val	B
UCHL1	3.40628688	6.414952251	5.563879642	0.000385535	0.029915675	0.480055665
FN3K	2.467110104	1.068581073	6.385600596	0.000143367	0.017147422	1.463110559
LRRC15	2.396603525	3.242246747	12.05935041	9.39095E-07	0.002040341	6.091559443
TMED3	2.348382189	0.267909408	9.646487836	5.85793E-06	0.003181835	4.501749371
PRXL2A	2.329353427	4.319409407	7.031837606	6.985E-05	0.012366311	2.167837575
GPNMB	2.246011912	5.923704358	8.156673501	2.22709E-05	0.006598267	3.265299132
PREX1	2.230033135	3.340390207	5.296220122	0.000542618	0.035725075	0.137647749
PDCD4	2.131433859	5.348675937	10.42454317	3.11964E-06	0.002751311	5.064153401
DPP4	2.124402099	4.092235778	6.703865192	9.99984E-05	0.015056145	1.817387429
ABI3BP	1.995009365	4.799140395	10.473617	3.00221E-06	0.002751311	5.09791653
FHOD3	1.990005852	3.541012099	5.680338032	0.000333289	0.027720397	0.625572825
NAMPT	1.947760731	10.48379892	10.1030109	4.02711E-06	0.002751311	4.838030789
MOCOS	1.912055668	2.432979806	5.11001281	0.000692363	0.041025672	-0.10716083
MAPK13	1.864321079	4.105202908	10.18060362	3.78407E-06	0.002751311	4.893389474
CORO2A	1.590543947	1.732680018	6.082457212	0.000204414	0.020497969	1.112164389
RIN2	1.538649074	2.578391616	6.232397612	0.00017127	0.018605626	1.287424196
MME	1.520542317	6.385428357	6.791493988	9.07471E-05	0.014083085	1.912451601
ANPEP	1.480114917	6.125246145	7.463548549	4.43566E-05	0.009799528	2.60751242
LCP1	1.457790325	7.095405373	8.479254642	1.64151E-05	0.005700157	3.552550521
NLRX1	1.42403614	2.804937103	7.582441566	3.92784E-05	0.009143457	2.724449861
IFI30	1.390291306	2.075204078	5.171572254	0.00063842	0.03959583	-0.02562653
OCIAD2	1.33445628	7.947795268	6.210885235	0.000175642	0.018767757	1.262481897
FCGRT	1.297022527	3.737062444	5.411976051	0.000467488	0.032835039	0.287098547
BNIP3L	1.296540884	3.436393145	5.044809155	0.000754924	0.043933907	-0.19417036
NDRG1	1.286541182	5.452779446	5.330472545	0.000519107	0.034881863	0.182088492
PTGES	1.25949649	7.282045048	6.254581295	0.000166886	0.01843664	1.313074131
ZBTB8OS	1.155655105	2.45397489	5.958338581	0.000237168	0.022038284	0.964569564
LRCH4	1.078391901	4.256657164	5.221308352	0.000598159	0.037852436	0.039813869
SDHAF4	1.061189655	2.928519247	5.244642831	0.000580225	0.037077522	0.070382649
CTSA	1.035207341	7.189140511	5.259162813	0.00056936	0.036743462	0.08936116
PDF	1.010904906	5.011488132	5.759187176	0.000302312	0.026627999	0.722915066
MAP3K4	0.965379352	2.323247732	5.115420816	0.000687432	0.041025672	-0.09997421
KATNA1	0.962989222	2.909962627	5.262107932	0.000567183	0.036743462	0.093206585
UPP1	0.962280291	6.16572353	5.596405953	0.0003701	0.02906403	0.520908166
ACP6	0.921517514	3.946919235	5.176747046	0.000634098	0.03959583	-0.01879971
LUZP1	-0.881415102	6.081180389	-4.997432187	0.000804207	0.04598089	-0.25780997
COL1A2	-0.932085114	5.505174627	-5.445635042	0.000447819	0.032651291	0.330163175
SPARC	-0.938986547	6.492589664	-5.297250246	0.000541894	0.035725075	0.138986958
FOXC2	-0.995759546	4.206808712	-4.973839255	0.000830036	0.047045011	-0.28963276
ANLN	-1.018739453	6.110447274	-5.523605429	0.000405622	0.030455612	0.429245433
NR2C2AP	-1.019363681	5.088940237	-5.022998593	0.000777194	0.044829669	-0.22342388
DUSP15	-1.029800687	6.312626243	-5.841792821	0.000273187	0.024392246	0.823878604
SP100	-1.038926125	5.724719407	-5.616600668	0.000360856	0.029037796	0.546190297
DNTTIP2	-1.054844562	4.943946195	-5.509904523	0.000412713	0.030568899	0.411903049
PDLIM5	-1.072816797	7.982646793	-5.663760081	0.000340232	0.027720397	0.60498564
PAGR1	-1.084707616	3.874922497	-5.401937246	0.000473534	0.032835039	0.274220365
HELZ2	-1.104312453	6.531982378	-6.049502751	0.0002126	0.020995844	1.073200457
INHBA	-1.108952549	5.511309209	-6.360752822	0.000147533	0.017171838	1.434846523
DSP	-1.119995032	7.502093555	-5.596824934	0.000369906	0.02906403	0.521433335
SFT2D3	-1.136706805	4.584165076	-5.709585003	0.000321414	0.027565467	0.661790224
KCTD3	-1.136784013	2.696888044	-5.431691843	0.000455856	0.032651291	0.312345088
LGALS1	-1.141122849	12.41998354	-4.932954339	0.000876943	0.049275137	-0.34498609
LIMA1	-1.14877935	7.714106248	-6.420725569	0.000137696	0.016934029	1.502913738
COL1A1	-1.164103404	7.195913852	-6.80482983	8.94233E-05	0.014083085	1.926826717
FN1	-1.170840064	8.218239558	-6.642423896	0.000107099	0.015056145	1.750100799
ISG15	-1.174742756	8.11188201	-6.557742216	0.000117807	0.015056145	1.656500739
STAT1	-1.179970343	7.678889126	-5.991731938	0.000227827	0.022038284	1.004504987
TAGLN	-1.182562671	10.256725	-5.150814967	0.000656083	0.03959583	-0.05305272
CCN2	-1.19275477	5.749879752	-6.558498873	0.000117706	0.015056145	1.657341535
TJP2	-1.196114432	6.947996395	-6.298065837	0.000158643	0.018141005	1.363145596
DNAJB4	-1.20611726	6.957932013	-5.698134158	0.000326008	0.027596334	0.647625903
DDAH1	-1.207835232	6.863218572	-5.158228705	0.000649715	0.03959583	-0.04324933
RHOBTB3	-1.209420945	2.619856275	-5.157298727	0.00065051	0.03959583	-0.0444786
TRIM14	-1.211940028	3.271581391	-6.108669203	0.000198148	0.020180109	1.143041422
CDCA3	-1.228357867	3.909002458	-5.716903311	0.000318515	0.027565467	0.670832224
GPATCH11	-1.234265937	4.187914622	-5.521874467	0.000406511	0.030455612	0.427056014
TNFRSF12A	-1.23664691	5.704634016	-6.839709701	8.60598E-05	0.014023443	1.964310201
HMGCS1	-1.23919106	7.013730843	-5.666334748	0.000339143	0.027720397	0.608185734
H1-3	-1.242381561	6.177893776	-6.111911238	0.000197387	0.020180109	1.146853394
AKIRIN2	-1.258088218	3.789628152	-5.404540045	0.000471958	0.032835039	0.277560847
PLSCR1	-1.258463637	5.984560566	-6.263079329	0.000165239	0.01843664	1.322881021
POSTN	-1.281355853	4.527814391	-6.601915992	0.000112082	0.015056145	1.705451915
MOCS2	-1.283515566	5.961313783	-5.524937857	0.00040494	0.030455612	0.430930448
ESM1	-1.318362849	4.7651276	-6.559957349	0.000117512	0.015056145	1.658961962
FGF1	-1.32567282	4.47288274	-7.295100349	5.28288E-05	0.010297518	2.438804354
PALLD	-1.329576194	6.652413035	-6.443358832	0.000134172	0.016817937	1.528468027
SPDL1	-1.343661023	5.012376887	-7.027211163	7.01985E-05	0.012366311	2.162994587
IL6ST	-1.34499114	5.028551138	-6.557885352	0.000117788	0.015056145	1.656659798
FAM107B	-1.360955987	4.925684269	-5.049671476	0.000750054	0.043933907	-0.18765892
NEXN	-1.381810701	6.576234259	-5.977445633	0.000231773	0.022038284	0.987440288
TUFT1	-1.384514511	3.897936694	-5.913565699	0.000250353	0.022663945	0.910762899
CCN1	-1.393540457	5.660429337	-7.415560288	4.66071E-05	0.009799528	2.559814859
TRAFD1	-1.444103277	2.809662459	-5.948286387	0.000240061	0.022038284	0.952515347
LHPP	-1.48015852	3.211624174	-6.654262024	0.000105689	0.015056145	1.763105917
IRF9	-1.485233946	4.913992299	-5.372434407	0.000491799	0.033742604	0.236282044
RHOB	-1.505524072	4.463967579	-7.311909872	5.19083E-05	0.010297518	2.455801405
ANAPC13	-1.507072581	2.663331372	-5.955112285	0.000238092	0.022038284	0.96070236
LMO7	-1.546730085	5.893236845	-8.525242141	1.57284E-05	0.005700157	3.592573693
GBP1	-1.553294342	5.57278384	-8.359405323	1.8365E-05	0.005700157	3.447167917
LXN	-1.564751686	8.517952637	-7.754088748	3.30387E-05	0.008613854	2.89021733
METAP2	-1.571327502	6.456813589	-7.808858311	3.12838E-05	0.00849616	2.942363033
IFIT2	-1.580110331	5.541249054	-6.133411366	0.000192425	0.020180109	1.172093897
THBS1	-1.582350156	4.979379583	-8.951462164	1.06767E-05	0.005353117	3.952918007
CREB3L1	-1.588317996	2.494210688	-6.579780311	0.000114912	0.015056145	1.680956467
SLC30A1	-1.588578261	4.293482106	-5.334753919	0.000516247	0.034881863	0.187630461
NMI	-1.59558396	5.000518677	-5.434657858	0.000454133	0.032651291	0.316137906
SPRY4	-1.624195002	3.709535593	-8.380315898	1.80072E-05	0.005700157	3.465667338
IFI35	-1.639643109	5.438177039	-7.612745172	3.80887E-05	0.009143457	2.753976099
CLSPN	-1.648882448	3.84185622	-7.046255381	6.87761E-05	0.012366311	2.182912008
SYNM	-1.660105632	4.029097368	-7.696438081	3.50037E-05	0.008775151	2.834939855
CBLL1	-1.713784738	2.992121469	-6.377610465	0.000144693	0.017147422	1.454031557
A2M	-1.788640965	10.64255809	-9.927470772	4.64321E-06	0.002751311	4.710890692
SAMHD1	-1.805076602	6.313532585	-8.102836884	2.3456E-05	0.006647236	3.216226684
RND3	-1.867388116	5.978197857	-9.983444804	4.43612E-06	0.002751311	4.751720536
IGFBP3	-1.890216566	4.558468478	-8.447167285	1.69138E-05	0.005700157	3.524489536
IFIT1	-1.952561426	7.60507316	-8.380014726	1.80123E-05	0.005700157	3.465401234
PRR3	-2.1040651	0.610996321	-7.428864674	4.59709E-05	0.009799528	2.573067586
FOSB	-2.149489401	2.855825165	-7.279214465	5.37152E-05	0.010297518	2.42270804
F3	-2.16638292	5.273551096	-11.22932903	1.69456E-06	0.002751311	5.594121212
FGD4	-2.190345548	3.954606882	-8.592799578	1.47763E-05	0.005700157	3.650958312
IFI44L	-2.300244951	4.238037241	-8.842375372	1.17724E-05	0.005480885	3.862480898
IFIT3	-2.300553796	6.796690377	-12.94821582	5.19013E-07	0.001691464	6.575070576
OAS2	-2.696676185	4.769662527	-6.934503921	7.75988E-05	0.013053435	2.065347725
HLA-B	-2.697771116	3.73465675	-6.928528891	7.81043E-05	0.013053435	2.059014927
PZP	-2.85978065	5.808634997	-10.6801399	2.55886E-06	0.002751311	5.237891926
CNN1	-2.896823587	6.061831738	-14.68687515	1.79884E-07	0.001172483	7.394923877

Supplementary Table S6. List of Transcription Factor activities inferred from differentially expressed proteins in T5H-FC#1-sh GPX1-05 vs. T5H-FC#1- sh Control conditions.

	statistic	source	condition	score	p_value	rnk
1	viper	AHR	t	-2.45075	0.014256	20
2	viper	AP1	t	-2.46448	0.013721	19
3	viper	AR	t	-2.95673	0.003109	11
4	viper	ATF3	t	-2.35352	0.018597	23
5	viper	CEBPD	t	-3.48787	0.000487	6
6	viper	CLOCK	t	-2.54327	0.010982	17
7	viper	E2F4	t	-2.42474	0.015319	21
8	viper	EP300	t	-2.49586	0.012565	18
9	viper	ETV4	t	2.289541	0.022048	7
10	viper	FOS	t	-3.22564	0.001257	7
11	viper	GATA6	t	-2.26678	0.023404	25
12	viper	HES1	t	-2.21279	0.026912	26
13	viper	HNF4A	t	-2.64982	0.008053	13
14	viper	IRF1	t	-3.72978	0.000192	5
15	viper	IRF2	t	-2.04926	0.040437	32
16	viper	IRF3	t	-2.02495	0.042872	34
17	viper	JUN	t	-2.60093	0.009297	15
18	viper	KAT7	t	-2.05399	0.039976	31
19	viper	MBD2	t	2.221783	0.026298	9
20	viper	MYC	t	-2.16161	0.030648	28
21	viper	NFKB1	t	-2.06231	0.039178	30
22	viper	NFKB2	t	-2.36445	0.018057	22
23	viper	NOTCH1	t	-2.20975	0.027123	27
24	viper	NR0B2	t	2.694022	0.00706	4
25	viper	NR1H2	t	3.587155	0.000334	1
26	viper	NR4A1	t	2.528495	0.011455	5
27	viper	NR5A1	t	-2.04667	0.040691	33
28	viper	POU5F1	t	-3.07125	0.002132	10
29	viper	PPARG	t	2.06311	0.039102	10
30	viper	RARB	t	-2.57668	0.009975	16
31	viper	REL	t	-2.87603	0.004027	12
32	viper	SMAD2	t	-4.65197	3.29E-06	2
33	viper	SMAD3	t	-4.15818	3.21E-05	4
34	viper	SMAD4	t	-3.19628	0.001392	8
35	viper	SRF	t	-3.12762	0.001762	9
36	viper	STAT1	t	-4.56595	4.97E-06	3
37	viper	STAT2	t	-4.87397	1.09E-06	1
38	viper	STAT3	t	-2.32048	0.020315	24
39	viper	STAT6	t	-1.96634	0.04926	35
40	viper	TAF1	t	-2.14527	0.031931	29
41	viper	TCF4	t	-2.63463	0.008423	14
42	viper	USF2	t	2.222409	0.026256	8
43	viper	YBX1	t	2.406485	0.016107	6
44	viper	YY1	t	2.751297	0.005936	3
45	viper	ZEB1	t	3.022993	0.002503	2

Supplementary Table 7. GSEA of differentially expressed proteins in T5H-FC#1-sh GPX1-05 vs. T5H-FC#1- sh Control conditions.

	Description	NES	p.adjust	Count	setSize	Gene_Ratio	core_enrichment
HALLMARK_OXIDATIVE_PHOSPHORYLATION	OXIDATIVE PHOSPHORYLATION	2.095819	1.27E-07	79	183	0.431693989	CPT1A/PHYH/MRPS12/NDUFB5/UQCR10/NDUFB4/COX6A1/NDUFC2/NDUFA3/COX7A2L/RETSAT/NDUFA9/TIMM50/NDUFS7/SUPV3L1/UQCRQ/CYC1/COX15/UQCRFS1/BAX/MGST3/NDUFA6/UQCRC2/MRPS22/ATP5F1C/OXA1L/SLC25A12/MFN2/LRPPRC/SDHD/COX6C/MRPL34/ATP5MF/COX7A2/TOMM22/ATP6V1C1/SLC25A4/NDUFB1/SLC25A5/UQCRC1/ATP6V1D/NDUFB6/NDUFB8/MRPS15/ATP5PB/ATP6V0C/ATP5ME/PDHB/VDAC3/SLC25A11/ATP5MG/CYB5R3/NNT/ATP6V1H/PDP1/COX4I1/VDAC1/SLC25A3/VDAC2/PDHA1/NDUFA7/ISCU/NDUFA4/ATP5PO/ATP5F1A/NDUFB3/ACAA1/MTX2/COX11/IMMT/MTRR/PHB2/SURF1/OPA1/TCIRG1/DLAT/RHOT2/PDHX/NDUFB7
HALLMARK_KRAS_SIGNALING_UP	KRAS SIGNALING UP	1.547136	0.045087	11	55	0.2	GPNMB/CTSS/LCP1/KIF5C/TMEM158/GNG11/DUSP6/GFPT2/FUCA1/PLAU/PLAUR
HALLMARK_P53_PATHWAY	P53 PATHWAY	1.503073	0.030156	31	96	0.322916667	HDAC3/IFI30/NDRG1/XPC/RRAD/STEAP3/UPP1/CYFIP2/EPHX1/PROCR/FUCA1/RETSAT/GM2A/BAX/COQ8A/SDC1/MAPKAPK3/RPS27L/ADA/POM121/IL1A/RB1/AK1/SEC61A1/RAP2B/CCP110/ISCU/PTPRE/TGFB1/PMM1/CD82
HALLMARK_APOPTOSIS	APOPTOSIS	-1.47264	0.044237	23	96	0.239583333	TOP2A/PEA15/DDIT3/ENO2/GPX1/LMNA/TAP1/TIMP3/MGMT/BCL10/SQSTM1/CASP3/IL1B/CTNNB1/CASP7/DPYD/IL6/H1-0/CASP2/IFITM3/CCND1/TNFRSF12A/RHOB
HALLMARK_MYC_TARGETS_V1	MYC TARGETS V1	-1.54574	0.006231	75	196	0.382653061	HNRNPR/EIF4H/KARS1/CDK2/DDX21/PPM1G/NPM1/RAD23B/EEF1B2/PSMA2/CLNS1A/VBP1/EIF3J/PSMA1/SNRPA/PABPC4/PSMB2/PSMA7/HNRNPD/PSMA6/TFDP1/PSMB3/RNPS1/UBE2L3/GSPT1/EIF1AX/SNRPD1/RANBP1/NOLC1/PABPC1/PSMA4/POLE3/STARD7/RRM1/SNRPD3/CSTF2/HNRNPA3/SRPK1/NOP16/KPNA2/TYMS/IMPDH2/BUB3/DHX15/COX5A/PCNA/LSM2/NME1/LDHA/LSM7/SRM/NHP2/PRPS2/SERBP1/SNRPD2/HNRNPA2B1/ERH/SRSF2/HPRT1/GLO1/EXOSC7/PA2G4/PPIA/PWP1/CNBP/RRP9/SRSF1/TRA2B/HNRNPA1/CDC20/PGK1/SRSF7/SRSF3/YWHAE/CCNA2
HALLMARK_ALLOGRAFT_REJECTION	ALLOGRAFT REJECTION	-1.56891	0.028196	14	64	0.21875	B2M/TAPBP/TAP1/BCL10/ICAM1/IL1B/HLA-A/HLA-E/IL6/TAP2/PSMB10/HLA-G/INHBA/STAT1
HALLMARK_G2M_CHECKPOINT	G2M CHECKPOINT	-1.63403	0.004564	51	148	0.344594595	MKI67/HNRNPD/TFDP1/PRC1/RPA2/GSPT1/SNRPD1/NOLC1/NOTCH2/EWSR1/CBX1/SS18/UBE2S/KPNA2/TPX2/UBE2C/TACC3/BUB3/HMGN2/UPF1/PAFAH1B1/E2F4/NUSAP1/SMAD3/CCNB2/POLE/MARCKS/SRSF10/CHAF1A/LMNB1/NCL/TOP2A/SRSF2/PML/CHMP1A/KIF4A/KIF23/SRSF1/TRA2B/JPT1/CDC20/PRMT5/INCENP/DKC1/AURKB/CCNA2/ATRX/DR1/CCND1/ABL1/AMD1
HALLMARK_TGF_BETA_SIGNALING	TGF BETA SIGNALING	-1.73591	0.027157	10	30	0.333333333	PPP1CA/PPM1A/SMAD3/TJP1/TGFBR1/WWTR1/CTNNB1/ARID4B/ID1/THBS1
HALLMARK_HYPOXIA	HYPOXIA	-1.75857	0.002001	40	105	0.380952381	HSPA5/PLIN2/ETS1/BGN/P4HA1/GLRX/TPBG/TES/PGM2/NAGK/PFKFB3/PKLR/NEDD4L/PYGM/MAFF/GRHPR/LDHA/LOX/DDIT3/ALDOA/ENO1/ENO2/STC2/FOXO3/DUSP1/ALDOC/CSRP2/PGK1/TGM2/TPI1/GAPDH/FOSL2/IL6/CCN2/COL5A1/VHL/CCN1/LXN/IGFBP3/F3
HALLMARK_TNFA_SIGNALING_VIA_NFKB	TNFA SIGNALING VIA NFKB	-1.94396	0.000263	26	81	0.320987654	TSC22D1/SMAD3/MAFF/MARCKS/CEBPB/DUSP1/REL/ABCA1/TAP1/ICAM1/SQSTM1/SLC2A6/IL1B/FOSL2/IL6/NFKBIE/PDLIM5/INHBA/DNAJB4/CCND1/IL6ST/CCN1/RHOB/IFIT2/FOSB/F3
HALLMARK_EPITHELIAL_MESENCHYMAL_TRANSITION	EPITHELIAL MESENCHYMAL TRANSITION	-1.95785	2.95E-05	44	121	0.363636364	CXCL8/CALU/QSOX1/DAB2/VCAN/GPX7/LOX/BASP1/LOXL2/ENO2/PPIB/DPYSL3/FSTL1/FBN1/ITGA2/TIMP3/TGM2/CALD1/EDIL3/GJA1/TPM4/COL1A2/SPARC/TPM2/FOXC2/IL6/COL5A2/THBS2/COL4A1/INHBA/LGALS1/COL1A1/FN1/TAGLN/CCN2/TNFRSF12A/POSTN/COL5A1/MEST/CCN1/TPM1/RHOB/THBS1/IGFBP3
HALLMARK_IL6_JAK_STAT3_SIGNALING	IL6 JAK STAT3 SIGNALING	-2.02519	0.00124	8	25	0.32	GRB2/IL1B/IL6/STAT1/TNFRSF12A/IL6ST/IRF9/A2M
HALLMARK_INTERFERON_ALPHA_RESPONSE	INTERFERON ALPHA RESPONSE	-2.34314	1.27E-07	32	65	0.492307692	TRIM5/EIF2AK2/RIPK2/MOV10/GBP2/UBE2L6/SAMD9L/TRIM21/PNPT1/PSMB8/PARP9/B2M/TAP1/HLA-C/SAMD9/OASL/IFI44/PSMB9/LAP3/IFITM3/HELZ2/ISG15/TRIM14/PARP14/PLSCR1/TRAFD1/IRF9/IFIT2/NMI/IFI35/IFI44L/IFIT3
HALLMARK_INTERFERON_GAMMA_RESPONSE	INTERFERON GAMMA RESPONSE	-2.5651	5E-09	40	103	0.388349515	PNPT1/MX2/PSMB8/PML/B2M/TAPBP/TAP1/ICAM1/OASL/IFI44/CASP3/LYSMD2/PSMB9/CASP7/HLA-A/LAP3/IL6/PSMB10/HLA-G/IFITM3/HELZ2/OAS3/BPGM/ISG15/STAT1/TRIM14/PARP14/PLSCR1/CD274/TRAFD1/IRF9/IFIT2/NMI/IFI35/SAMHD1/IFIT1/IFI44L/IFIT3/OAS2/HLA-B
